# Multifunctional scalable coated paper sheets for UV shielding and sublimation printing applications

**DOI:** 10.1038/s41598-025-08734-4

**Published:** 2025-07-11

**Authors:** Abeer M. Adel, Fatma N. El-Shall, Mohamed A. Diab

**Affiliations:** 1 Cellulose and Paper Department, National Research Centre, 33El-Bohouth St. (Former El-Tahrir St.), P.O. 12622, Dokki, Giza, Egypt; 2 Dyeing, Printing and Textile Auxiliaries Department, National Research Centre, El-Buhouth St. 33, Dokki, Cairo, 12622 Egypt

**Keywords:** Silica-based cellulose nanofibers (SCN_NP_), Silica nanoparticles (SiO_2NP_), Paper coating, UV protection, Sublimation printing application, Environmental sciences, Materials science, Nanoscience and technology

## Abstract

Agricultural residues are produced annually; recycling these wastes in various ways is considered economically valuable. In this context, biopolymer-reinforced composite materials were developed to create alternative, eco-friendly, and sustainable resources for different applications. With advancements in innovative chemical techniques, cellulose nanofibers with silica have been simultaneously obtained. Rice residues were transformed into silica-based cellulose nanofibers (SCN_NP_) through hydrolysis using ammonium persulfate (APS) under microwave radiation at 70 °C, 1.25 M APS, an irradiation time of 20 min, and a liquor ratio of 1:75. Additionally, rice residue was converted into silica nanostructure SiO_2NP_ via hydrochloric acid hydrolysis followed by calcination at 600 °C. The principal characterizations of the extracted SCN_NP_ and SiO_2NP_ were evaluated using FTIR, XRD, BET surface area analysis, SEM, TEM, EDX and ζ-potential measurements. To produce cellulose/silica hybrid composites on a paper matrix, co-processing of the isolated SiO_2NP_ and/or SCN_NP_, which contained silica, was considered. Different concentrations of [SiO_2NP_ (0.25–3%w/v)/SCN_NP_ (0.5%w/v)] nanocomposites were used to modify the fabricated paper sheets, with cationic polyacrylamide (CPAM) serving as a binder. Fabricated paper sheets treated with various concentrations of (CPAM/SiO_2NP_/SCN_NP_) nanocomposite solutions were prepared. The impact of SiO_2NP_ and/or SCN_NP_ on the modified paper’s surface structure, strength, barrier, and UV shielding characteristics was examined. To evaluate color properties, the fabricated paper sheets treated with different concentrations of CPAM/SiO_2NP_/SCN_NP_, were silk-screen printed using disperse dye. Under different conditions (temperatures of 170–210 °C and time of 30–60 s.), the printed paper sheets were tested as heat transfer paper in sublimation transfer printing of polyester fabrics. Polyester samples printed using sheets treated with CPAM/0.5% SCN_NP_ and CPAM/3% SiO_2NP_ showed enhanced color depth. All polyester samples printed with modified sheets demonstrated outstanding fastness properties. Additionally, some treated paper sheets showed remarkable transfer stability during a second printing run.

## Introduction

Regular exposure to light, particularly UV light, triggers photolysis and photooxidation reactions that degrade food quality. These processes accelerate food degradation by generating free radicals and reactive oxygen species, which impart unpleasant tastes and odors, reduce nutritional content, and cause discoloration^1,2^. Ultraviolet (UV) radiation, a form of electromagnetic radiation with wavelengths shorter than visible light (100–400 nm), is categorized into three bands based on wavelength: UVC (200–280 nm), UVB (280–315 nm), and UVA (315–400 nm)^3,4^. UV exposure not only promotes food spoilage but also increases the risk of microbial contamination by pathogens like E. coli and L. monocytogenes, leading causes of foodborne illnesses^5,6^. Globally, over 550 million food-borne illnesses occur annually, primarily due to contaminated food consumption^7,8^. To address the environmental and health issues posed by non-biodegradable plastic packaging, researchers have turned to biopolymer-based UV protective films derived from natural materials such as (chitosan, cellulose, starch, gelatin, and alginate …etc.)^9,10^. These biopolymers are non-toxic nature, biocompatible, and biodegradable, making them sustainable alternatives to synthetic polymers^11,12^. Agricultural waste management is critical for sustainable resource use, given the 30 to 35 million tons of residue produced annually^13,14^. Traditionally, these wastes (e.g., sugarcane bagasse, wheat straw, rice husks, and corn stalks) were burned or composted, but recent efforts have focused on converting them into value-added products like nanocellulose (NC)^15^. A plentiful natural biopolymer, cellulose can be efficiently made into hydrogel and composite forms^16,17^. NC is renewable, non-toxic, and biodegradable, with exceptional mechanical properties, a large surface area, high crystallinity, and low density^18^. Ammonium persulfate (APS), an eco-friendly oxidant, enables efficient NC production via microwave-assisted synthesis—a rapid, single-step process^19,20^. However, NC’s poor UV-blocking and thermal stability limits its packaging applications. To overcome this, nanomaterials like SiO₂, ZnO, TiO₂, Cu NPs, CeO₂, carbon quantum dots (CQDs), and plant extracts are incorporated for enhanced UV protection^1^. While paper and paperboard are widely used in packaging, their low mechanical strength, high water vapor penetration, and poor barrier capabilities restrict their effectiveness. By incorporating nanoparticles (e.g., SiO_2_, ZnO, and TiO_2_)^21,22^ into paper matrix formulations, these materials gain improved gas barrier properties, UV resistance, and antimicrobial/antioxidant functionality, extending food shelf life^,^^23–25^.

Rice husk ash (RHA), a byproduct of combustion, is rich in silica (SiO_2_), a chemically, physically, and thermally stable, and cost-effective material^26^. With 760 million tons of rice husk waste and 1.14 billion tons of rice straw waste generated annually (containing 15–20% silica), extracting silica nanoparticles (SiO_2NP_) offers a sustainable solution^27,28^. Incorporating SiO_2NP_ and/or silica-based cellulose nanofibers SCN_NP_ into paper coatings improves tensile strength, water resistance, UV blocking, and food preservation^29–31^.

In transfer printing, once the printed paper and fabric come into direct contact within a press or calendar at an elevated temperature, specific nonionic dispersed dyes are transferred in the vapor phase from a thermoplastic polymer film to the interior of the fabric surface adjacent to the printed paper. This process occurs through two complementary stages: first, a pattern is printed on special transfer paper using an appropriate printing technique; second the pattern is heated, causing the dye vapor to diffuse from the paper into the fabric using a flat-bed press. The dye vapor then penetrates the fiber pores and becomes permanently embedded within the fibers^32^. Digital printing (including Inkjet, Transfer, and Laser) necessitates inks with outstanding fastness properties, and the printed paper should be subjected to per-finishing treatment to remain durable. The resolution of the printed image is controlled by the coating layer on the paper surface. Parameters involved surface area, pore structure, and SiO_2NP_ additive mainly affect image properties. Applying a thin layer of SiO_2NP_ coating to the paper surface can improve fastness by decreasing coating permeability, which in turn reduces printing ink dispersion on the paper surface and structure^33–35^.

Our study had several main objectives. First, we aimed to develop an efficient single-step chemical procedure, using microwave irradiation, to extract cellulose nanofibers (CN) from rice waste along with silica, resulting in a silica-based cellulose nanofibers (SCN_NP_) hybrid nanocomposite. The second step involves creating novel nanocomposites using locally accessible raw materials. To achieve this, pure SiO_2NP_ was extracted from rice husk (RH) and combined with hydrophilic polymers, including SCN_NP_ to enhance the properties of nanocellulose. We investigated the potential advancement of paper uses and applications by improving the chemo-physical characteristics of these hybrid organic/inorganic nanocomposites and leveraging renewable resource-based technologies. Third, to obtain multifunctional applications in packaging and printing, SiO_2NP_ and/or SCN_NP_ were utilized as coating materials to enrich the surface of paper sheets, and their mechanical, barrier, and UV radiation shielding properties were then evaluated. Fourth, the sublimation transfer printability of treated paper sheets was examined in the printing of synthetic fabrics (polyester) under various conditions (temperatures and times), and fastness measurements (color strengths K/S, washing, light, and perspiration) were assessed using standard methods.

## Experimental

### Materials

The unbleached pulp made from rice residue was purchased by the Rakta Company for pulp and paper in Egypt. The chemical composition of rice agro-waste pulp was checked according to the Tappi standard method, including silica at 13% (T 245 om-94), lignin at 14% (T 222 om-88), ash at 15% (T 211), pentosane at 15% (T 223 cm-84), and α-cellulose at 55% (T 203 cm-99). We purchased ammonium persulfate (APS) from Sigma-Aldrich. Every test was run in triplicate, and the data’s mean value was emphasized.

### Extraction of SCN_NP_ and SiO_2NP_ nanoparticles

#### Silica-based cellulose nanocrystalline (SCN_NP_) isolation with microwave support

**Fig. 1:**
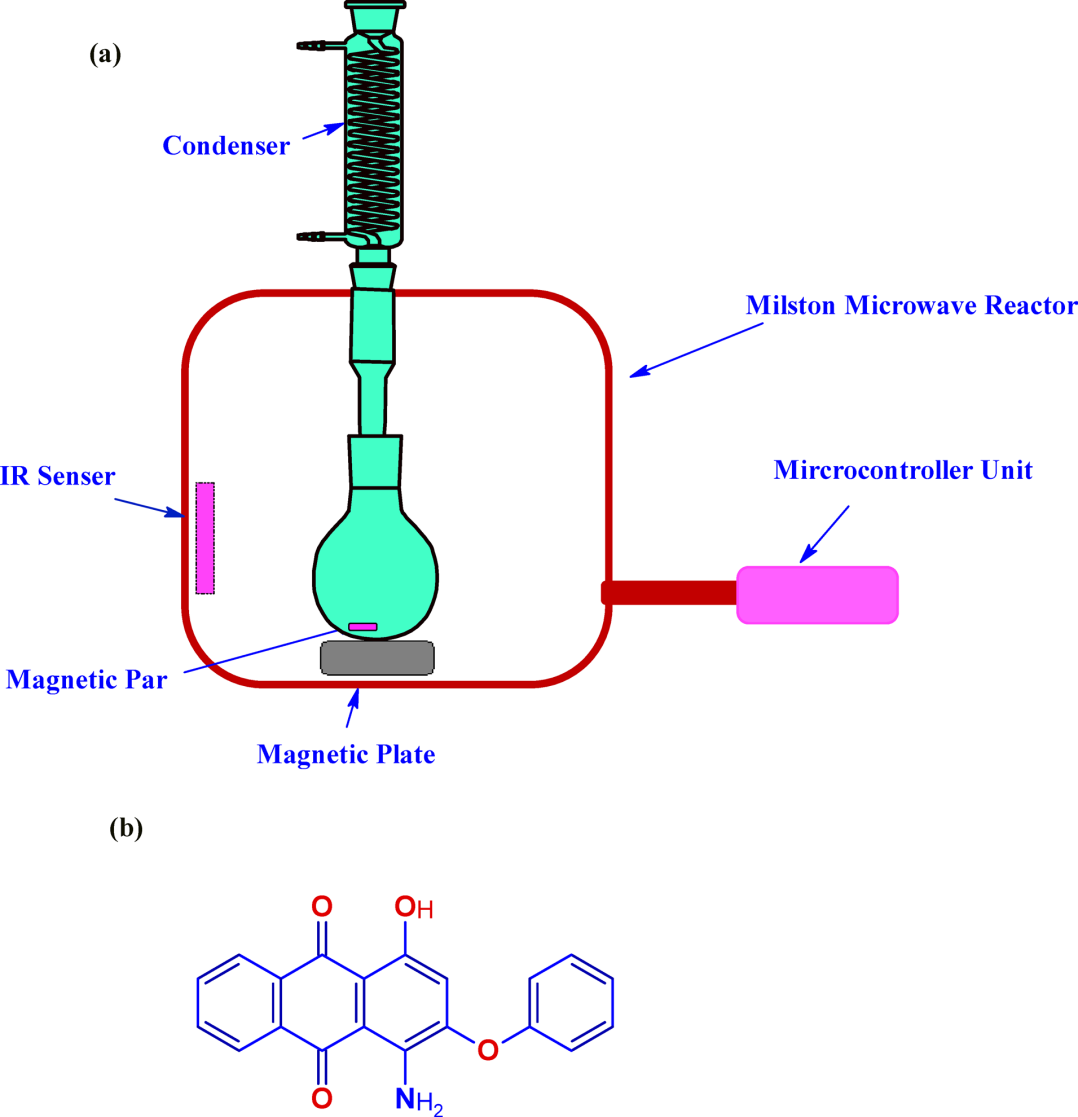
Schematic diagram of the modified microwave reactor system used in in SCN_NP_ preparation (**a**) and Chemical structure of Disperse Red 60 (**b**).

In the strategy to prepare SCN_NP_ from rice residue, the following parameters were used: temperature (70 °C), concentration (1.25 M of APS), irradiation time (20 min), and liquor ratio (1:75). The reaction mixture for one gram of rice waste fiber was sterilized in a completely hermetic microwave reactor coupled to a perpetually cooling system. Dialyzing the resulting suspensions against distilled water for four to seven days was necessary to reduce the solution conductivity to approximately 5 μscm^−1^ (pH 4). The yield of the prepared SCN_NP_ was calculated as (27.93 ± 0.55%), which was determined by subdividing the weight of the final lyophilized product by the original weight of the starting material. The schematic diagram of the modified microwave reactor system used to extract SCN_NP_ is presented in Fig. 1a.

#### Pure silica nanostructure separation from rice husk

RHA with a high silica concentration was produced when the rice husk RH was ignited. RH was utilized as a cost-effective and abundant source of biogenic silica nanoparticles for obtaining pure SiO_2NP_ with regulated phase, purity, and shape. This was accomplished by calcining RH in an oven muffle at 600 °C after pre-treating it with hydrochloric acid (1 M HCl). Subsequently, SCN_NP_ were separated from RHA as sodium silicate using sodium hydroxide (2 M NaOH). The silicate solution was then titrated with hydrochloric acid (2 M HCl) until the pH reached 7.

The following is the proposed mechanism for alkali/acid reaction: To extract silica, RHA was first dissolved in sodium hydroxide and then converted into sodium silicate according to Eq. (1). The second step is the precipitation using hydrochloric acid, as shown in Eq. (2):

1 $${\text{2NaOH}} + {\text{xSiO}}_{{2}} \to {\text{Na}}_{{2}} {\text{SiO}}_{{3}} + {\text{H}}_{{2}} {\text{O}}$$

2 $${\text{Na}}_{{2}} {\text{SiO}}_{{3}} + {\text{2HCl}} \to {\text{SiO}}_{{2}} \left( {{\text{gel}}} \right) \downarrow + {\text{2NaCl}} + {\text{H}}_{{2}} {\text{O}}$$

The establishment of a prolonged 3D Si–O–Si network results from the alkaline treatment, followed by hydrochloric acid solubilization of the supported silanol (R_3_Si–OH) groups and subsequent condensation^28^.

### Preparation of paper sheet

The paper sheet was prepared from bagasse agro-waste. The fibers underwent two chemical processes, lignin removal and cellulose isolation. Using the S.C.A. sheet old model (AB Worentzen and Wettre), the paper sheet was made in accordance with the S.C.A. standard. Within the apparatus, a sheet with a diameter of 165 mm and a surface area of 214 cm^2^ was created. Approximately 1.8 g of oven-dried pulp was used for each sheet. After formation, a hydraulic press was used to press the sheet for four minutes, and it was dried with the aid of a rotating drum.

### Fabrication of SiO_2NP_ /SCN_NP_ nanocomposite on paper sheets

The SiO_2NP_/SCN_NP_ nanocomposite was used to coat the prepared sheets, and cationic polyacrylamide (0.2% w/v CPAM) was used as a binder. To ensure the homogeneity of the solutions, the mixtures were stirred with a homogenizer for 3 min, followed by sonication for 15 min. The fabricated paper sheets were then treated with varying concentrations of the (CPAM/SiO_2NP_/SCN_NP_) nanocomposite solutions using a coating applicator, as indicated in Table 1. The final coating thickness was approximately 120 μm. Subsequently, the paper sheets were hard-pressed between two sheets of filter paper and then dried on a cylindrical drum at 105 °C for two hours. Prior to examination, the treated paper samples were conditioned at 50% relative humidity and 23 °C for 24 h.

**Table 1:** Different concentration for (CPAM/SiO_2NP_ and/or SCN_NP_) nanocomposite solutions.

Paper sheet samples	CPAM% w/v	SiO_2NP_% w/v	SCN_NP_% w/v
S_0_	-	-	-
S_1_	0.2	-	-
S_2_	0.2	-	0.5
S_3_	0.2	0.25	0.5
S_4_	0.2	0.5	0.5
S_5_	0.2	1.00	0.5
S_6_	0.2	3.00	0.5
S_7_	0.2	0.25	-
S_8_	0.2	0.5	-
S_9_	0.2	1.00	-
S_10_	0.2	3.00	-

### Characterization and chemical composition of SiO_2NP_, SCN_NP_, and modified paper sheets

The following measurements were used to characterize the extracted SCN_NP_, SiO_2NP_, and modified paper sheet chemically and morphologically:

FTIR Spectroscopy: The FTIR spectra of the variously produced samples were recorded using FTIR spectroscopy (JASCO FTIR 6100 spectrometer, Tokyo, Japan) in the 4000–400 cm^-1^ region, with 60 scans and a resolution of 4 cm^-1^.

X-ray Diffraction (XRD): An X-ray diffractometer (Siemen D5000) with a CuKα radiation (λ = 1.5406 Å) running at 30 nA and 40 kV was monitored to perform an XRD examination. Transmission Electron Microscopy (TEM): The high-resolution JEOL-JEM 2100 (Tokyo, Japan) was used to conduct TEM examination.

Energy-Dispersive X-ray Spectroscopy (EDAX) and Environmental Scanning Electron Microscopy (ESEM): A Quanta FEG-250 microscope (Waltham, MA, USA) was used to perform EDAX and ESEM on customized paper sheets at a voltage of 20 kV.

Particle Size and Porosity of SiO₂_NP_: the average particle size and porosity structure of SiO_2NP_ were determined using the nitrogen adsorption–desorption isotherm and the Quanta Chrome Touch Win device.

Surface Area Estimation: The density functional theory (DFT), Langmuir technique, and Brunauer–Emmett–Teller (BET) methods were employed to estimate the average surface area using a particle sizing system (Santa Barbara Inc., California, USA).

Thermogravimetric Analysis (TGA) and Derivative Thermogravimetry (DTG): The TG and DTG curves for coated paper sheets were investigated using a PerkinElmer AC7/DX TGA7 thermal analysis controller. The tests are conducted in a dynamic nitrogen environment (20 ml/min) with a heating rate of 10 °C/min in a temperature range of 20–600 °C using platinum crucibles.

Ultraviolet Protection Factor (UPF) Estimation: The UPF was estimated using a UV-Shimadzu 3101-PC-Spectrophotometer in accordance with the Australian/New Zealand Standard (AS/NZS-4399-1996).

### Strength and barrier characteristics of paper sheet

A universal testing machine (LR10K; Lloyd Instruments, Fareham, UK) was utilized to measure tensile strength by applying a 100-N load cell at a constant crosshead speed of 2.5 cm/min in accordance with TAPPI standard procedure (T494-06). ASTM E96-E80 was followed while determining water vapor permeability (WVP). Five grams of anhydrous calcium chloride were placed in aluminum cups, which were conditioned for 24 h at 25 °C and 50% relative humidity before being hermetically covered with coated paper sheets. The coated side faced the humidified side, and The WVP was established using the following formula:

3 $${\text{WVP}} = {\text{Wx}}/{\text{tA}}\,\Delta {\text{P}}$$

where x is the film thickness, A is the permeation area, (W/t) is the slope of the weight loss over time, and ΔP is the partial water vapor pressure difference between the atmosphere in the cup and the saturated sodium chloride solution, corresponding to 0–75% relative humidity (i.e., 2.385 kPa).

The ASTM D722-93 procedure was used to determine oil resistance. Five grams of sand with a specific particle size and a piece of white book paper were placed on top of the treated paper samples. The sand was saturated with a specified quantity of oil that had been combined with soluble red dye. Following that, the treated paper sheet test area was subjected to conditions of 50% relative humidity and 25 °C. The time required for oil penetration, observed as the red staining of white book paper through the modified paper samples, was recorded to the nearest ten seconds. For each measurement, the mean value of three estimations was determined, and the standard deviations (STDEV) were calculated.

### Printing process and color strength measurements

The 150 g/m^2^ polyester fabric (provided by a private Egyptian company) was treated with 0.5 g/l anionic detergent and 1 g/L sodium carbonate for one hour at 70 °C, then allowed to air dry at room temperature. The thickening agent Bercolin CPK was provided by Berssa-Turkey, the dispersed dye Disperse Red 60 (200% LS) Fig. 1b was provided by Zhejiang Runtu Co., Ltd, and the 60-gm transfer paper was provided by Protucal Soporcel Company.

#### Printing paste and printing technique

Here is the recipe for the paste listed in order: 2:4:94 water, synthetic thickener, and dispersed dye. The treated and commercial papers are printed using the prior recipe and then air-dried using a silk screen process. Additionally, the previously printed paper is used to print the polyester fabric via a heat transfer technique. A hydraulic heat press machine (40 × 60 cm) was used for the transfer. The temperatures and timeframes tested for the transfers were 170, 190, and 210 °C, and 30 and 60 s, respectively. The printed samples were allowed to cool at room temperature before the paper is removed.

#### Color strength (K/S) and CIELAB color parameters.

The Hunter Lab (Ultra scan-PRO D65, USA) evaluates the color strength and CIELAB color parameters (L*, a*, and b*) of the prints at λ_max 680_ (nm). The Kubelka–Munk relationship was used to quantify the color strengths (K/S) of printed samples.

4 $${\text{K}}/{\text{S}} = \left( {1 - {\text{R}}} \right)^{2} /2{\text{R}}$$

where S is the scattering coefficient, K is the absorbance coefficient, and R is the reflectance^36^. The sample’s brightness is indicated by L*, and its red-green and yellow-blue shifts are shown by a* and b*. The fastness properties to washing, perspiration, and light were evaluated using the standard procedures outlined in the AATCC Technical Manual, Test Methods 8 (1989) 68, 23 (1993); 15 (1989) 68, (1993) 30 and (16-2004), respectively.

## Results and discussion

### Microwave treatment mechanism

The interaction was based on the scientific theory that microwave radiation has high selectivity, and this selectivity depends on the dipole moment of the materials exposed to it. Furthermore, numerous investigations have demonstrated that microwave irradiation superior to conventional heating in terms of enhancing the efficacy of persulfate ions in APS compounds. Thermal and non-thermal effects, rapid and even heating, and polar coupling are the reasons for the high rate of sulphate radical production when combining microwave and APS processes. Water is the perfect solvent for processes aided by microwaves because of its high polarity and the great efficiency of persulfate ions when exposed to microwave radiation. The fundamental concept for producing high-quality SCN_NP_ is the proper ratio of salt to water, temperature, and energy^20^. Hydrogen peroxide molecules and sulphur tetraoxide free radicals were produced when the APS aqueous solution was exposed to microwave radiation. These hydrogen peroxide molecules and sulphur tetraoxide free radicals have the ability to hydrolyze cellulose’s amorphous portions and open the aromatic rings of lignin, producing SCN_NP_^37^.

5 $$\left[ {{\text{O}}_{{3}} {\text{SO}}{-}{\text{OSO}}_{{3}} } \right]^{{2}} - + {\text{ Microwave}}\;{\text{irradiation}} \to {2}\left[ {{\text{SO}}_{{4}} } \right]^{ \cdot }$$

6 $${\text{S}}_{{2}} {\text{O}}_{{8}}^{{2-}} + {\text{ 2H}}_{{2}} {\text{O}} \to {\text{2HSO}}_{{4}}^{ - } + {\text{H}}_{{2}} {\text{O}}_{{2}}$$

### Silica-based cellulose nanocrystals (SCN_NP_) and silica nanoparticles (SiO_2NP_) characterization

#### FTIR analysis

**Fig. 2:**
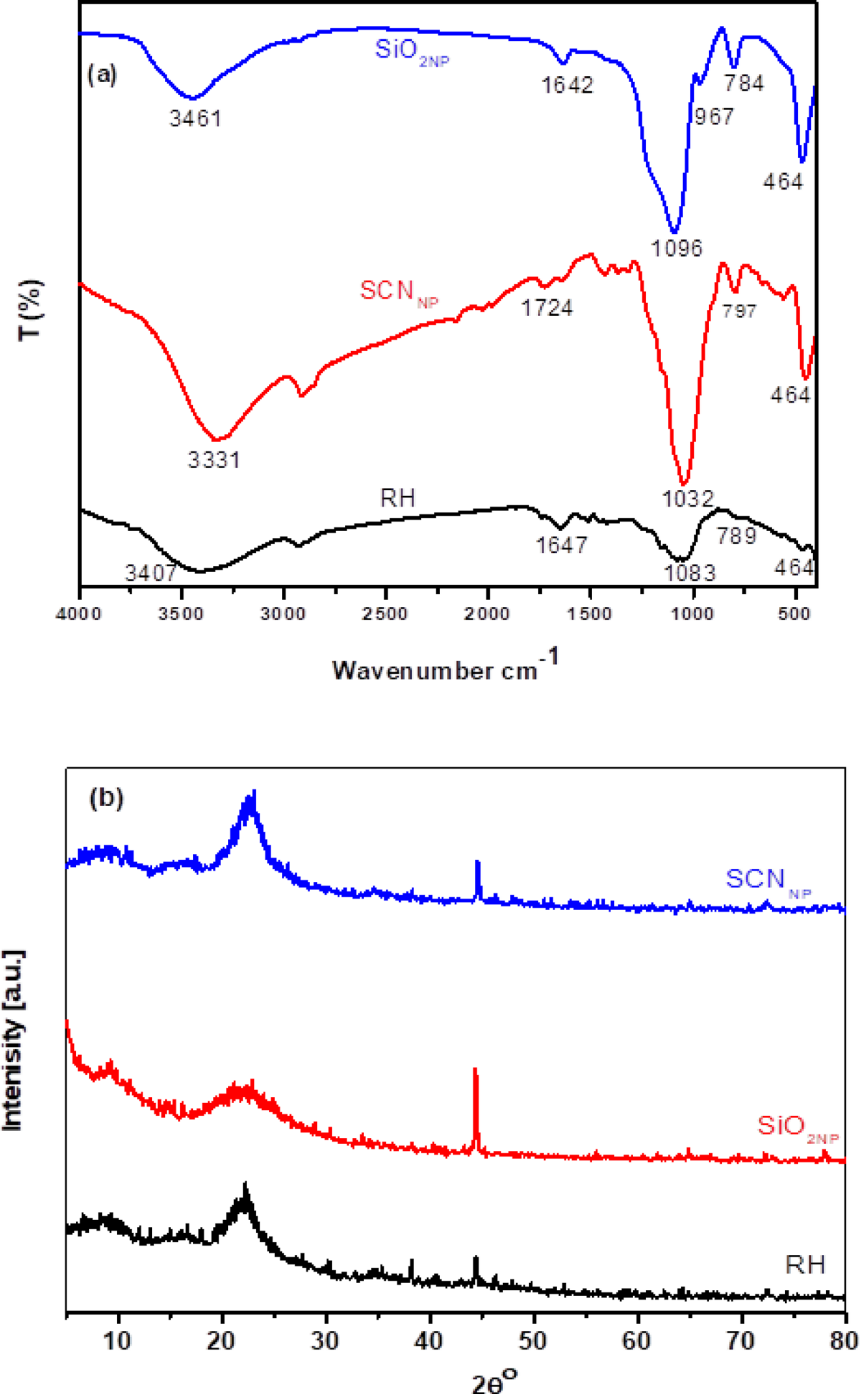

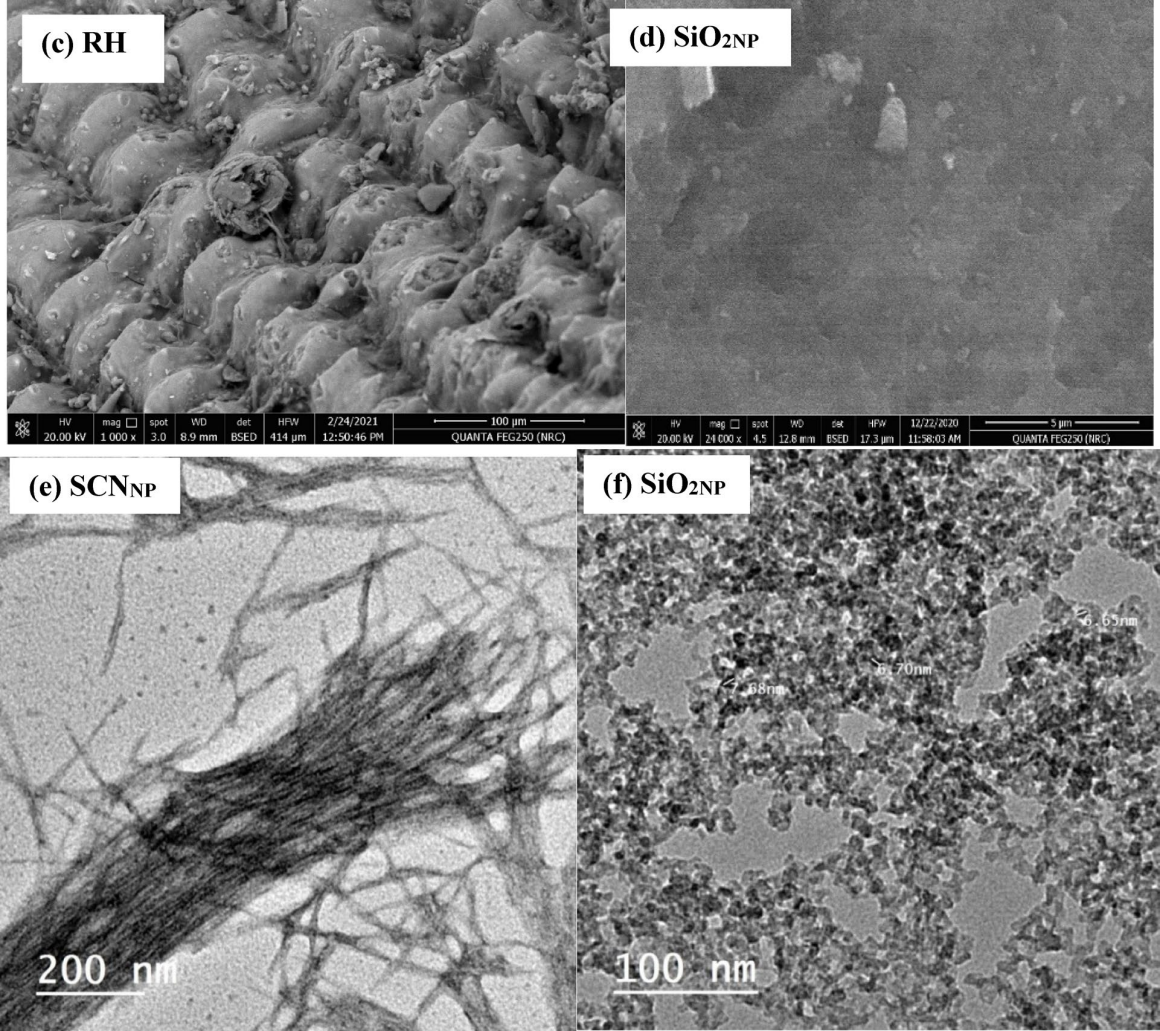

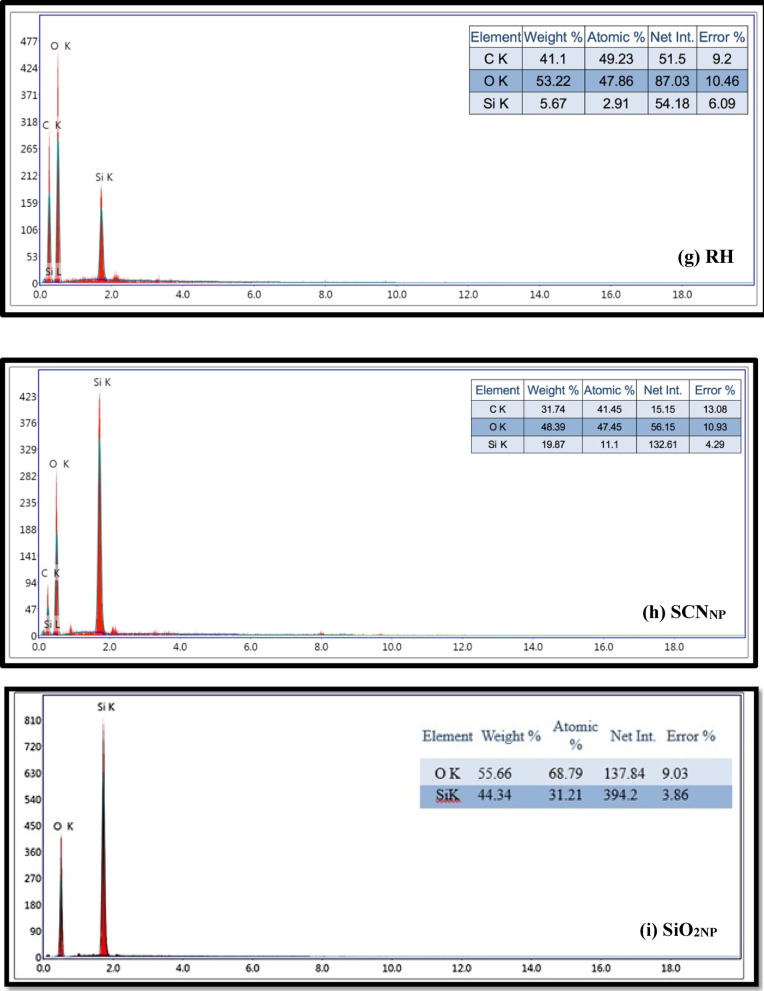

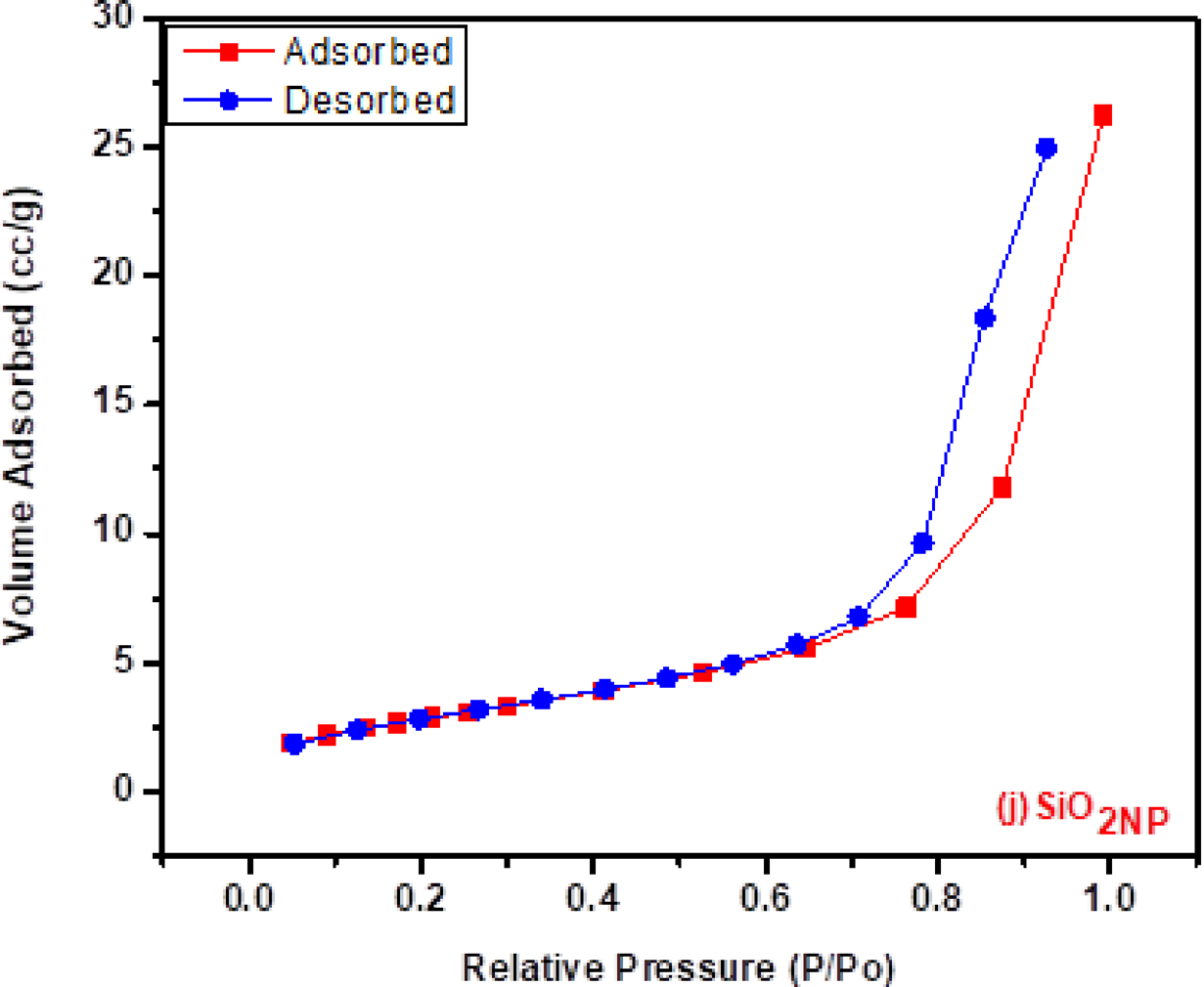
FT-IR spectra of RH, SCN_NP_ & SiO_2NP_ (**a**); XRD profiles of RH, SCN_NP_ & SiO_2NP_ (**b**); SEM photos for RH (**c**) & SiO_2NP_ (**d**); TEM micrographs of SCN_NP_ (**e**) & SiO_2NP_ (**f**); EDX analysis for RH (**g**), SCN_NP_ (**h**) & SiO_2NP_ (**i**) and isothermal nitrogen adsorption/desorption micrographs (**j**) for extracted SiO_2NP_.

As seen in Fig. 2a, FTIR analysis was applied to trace the molecular structure of the raw RH, produced SCN_NP_, and SiO_2NP_ employed in this investigation. Numerous distinctive absorption peaks were visible in the raw RH spectra, indicating the presence of inorganic materials, such as SiO_2NP_, and organic materials, including cellulose, hemicellulose, and lignin. The broad peak in the RH spectrum at approximately 3407 cm^-1^ represents the O-H stretching vibrations. In the structure of cellulose, hemicellulose, and lignin, the symmetric and asymmetric stretching vibrations of the C–H aliphatic bonds in the –CH_3_ and CH_2_ groups can be linked to the absorption peak at 2914 cm^-1^. The vibration bands at 1460, 1424, and 1370 cm^-1^ are attributed to the C-H stretching vibrations of the CH_2_ groups. In addition to the vibration brought on by adsorbed water molecules, the peak at 1647 cm^-1^ is attributed to the stretching vibrations of the -C=O groups in aldehydes and ketones, whereas the peak at 1515 cm^-1^ is attributed to the stretched -C-O groups in carboxylates. The peak at 1159 cm^-1^ represents the C-O stretching of the aliphatic ether. Furthermore, the stretching vibrations of the Si-O-Si groups’^38^ manifest as broad peaks at 1083, 789, 523, and 465 cm^-1^.

For cellulose nanocrystals made utilizing the microwave irradiation method (SCN_NP_), FTIR not only shows the entry of new bands but also changes in the intensities and locations of the key cellulose fingerprint peaks (Fig. 2a). The peak from 2900 cm^-1^ to 2850 cm^-1^ corresponds to the stretching vibrations of the methyl groups, whereas the broad band in the area around 3314 cm^-1^ is ascribed to the intra and inter-hydrogen bonded O–H stretching vibrations. The deformation vibration of adsorbed water molecules was represented by the bands at approximately 1630 cm^-1^. The glycosidic ring vibrations of C–O–C, C=O, C–H, and O–H are responsible for the distinctive cellulose fingerprint bands, which are found in the 1500–800 cm^-1^ spectral range^39^. A corresponding curve for the carboxyl group appeared at 1705 cm^-1^ and 1750 cm^-1^ peaks for SCN_NP_, which could be interpreted as the -C=O valence vibration of -COOH groups through the interaction of cellulose fibers with free radicals created during the chemical treatment under microwave irradiation^37^. The carbonyl groups in aldehydes and ketones’ –C=O stretching vibrations were identified as the cause of the peak at 1648 cm^-1^. Furthermore, we may infer from the increase in peak intensity at 1032 cm^-1^, which corresponds to the stretching of the pyranose ring from -C-O-C- that cellulose content and crystallinity rise while the pyranose ring stays intact with APS treatment. Additionally, the decrease in the band’s intensity at 900 cm^-1^ due to asymmetric out-of-plane ring stretching in cellulose caused by the β-linkage, which represents the amorphous portion of the cellulose fibers, shows that the microwave-prepared SCN_NP_ retains a high degree of crystallinity^40,41^. Two distinctive vibrational peaks for Si-O-Si also emerged at wavenumbers ≈ 797 cm^-1^ and 464 cm^-1^. Furthermore, using microwave radiation, the extent of oxidation was calculated by analyzing the infrared spectra of the generated SCN_NP_ samples, which reached 0.076. The degree of oxidation concerning the cellulose backbone structure can be determined by^26^ comparing the intensity of the carboxylate peak at 1750 cm^-1^ to that of the strong band near 1060 cm^-1^.

The FTIR spectrum of the SiO_2NP_ silica gel, shown in Fig. 2a, showed vibrational bands characteristic of amorphous silica and adsorbed water molecules at approximately 464, 967, 784, 1096, 1543, 1642, 2930, and 3461 cm^-1^. The bending and stretching vibrational bands of the adsorbed water molecules appeared around 1642 and 3461 cm^-1^, respectively, consistent with the previously reported findings^42,43^. The peaks at 1096, 967, 784, and 464 cm^-1^ were attributed to the siloxane group stretching vibrations within the synthesized SiO_2NP_. Specifically, the vibrational absorption at about 1096 cm^-1^ corresponded to the siloxane (Si-O-Si) linkages of the silicate network. Furthermore, symmetric and asymmetric vibrational bands of the Si-O-Si bonds were observed around 784 and 967 cm^-1^, respectively. The broad vibrational band at 3461 cm^-1^ was due to the O-H stretched vibration of the silanol groups (Si-OH) present in the silica gel nanoparticles. These findings are consistent with the published data and corporate to the amorphous chemical structure of SiO_2NP_'s^44^.

#### X-ray diffraction

XRD analysis of RH was performed to determine whether the investigated materials were amorphous or crystalline. Figure 2b shows the main diffraction peaks for RH at Bragg angles (2θ) of 15.5° and 22.33°, which are attributed to the diffraction patterns from the crystalline structure of its cellulose content. A peak equivalent to silica, appeared at Bragg 2θ angles at 44.48°.

The impact of the microwave irradiation approach on APS hydrolysis and the crystalline structure of cellulose were demonstrated by the XRD profiles of SCN_NP_ fibers (Fig. 2b). The crystalline form of cellulose I exhibited four lattice peaks at 2θ = 14.86 ($\overline{110}$), 16.08 (110), 22.58 (200), and 34.18 (004). Upon separation of the silica-based cellulose nanocrystals (SCN_NP_), a peak corresponding to silica observed at a Bragg 2θ angle of 44.48 (212). Additionally, the amorphous portions of the cellulose fibers, as well as the non-cellulosic materials (lignin, hemicellulose, and extractives) were eliminated. The crystalline regions underwent rearranged into a more ordered structure, resulting in a crystalline index of 66.2% for the SCN_NP_ sample produced using microwave radiation. This outcome attests to the effectiveness of the developing procedure utilizing the suggested approach^13,45^.

XRD profiles for a sample of SiO_2NP_ derived from gray RHA at 600 °C are displayed in Fig. 2d. These profiles suggest that burning the hulls at 600 °C resulted in the formation of an amorphous biogenic SiO_2NP_ structure. The primary peaks of SiO_2NP_ were observed at Bragg 2θ angles of 22.18° and 44.39°, corresponding to the (101) and (212) planes, respectively^46^. The broadness of the XRD peaks of the extracted SiO_2NP_ indicates that the synthesized biogenic silica nanoparticles were amorphous and nanoscale in dimension. Furthermore, the acid treatment altered the chemical composition of the silica nanoparticles. The sharpness and high intensity of peak at Bragg 2θ 44.39 for SiO_2NP_, following the HCl acid treatment process, indicates the high purity of extracted silica due to the removal of oxide impurities. It is known that the calcination temperature influences the type of silica produced with crystalline forms typically developed above 700–900 °C^47^.

#### Morphological study

The microstructure of the external surface of RH and SiO_2NP_ was observed using SEM, as shown in Fig. 2c, d. The surface morphology of RH revealed well-arranged micro-bumps across its surface. The RH outer epidermis exhibited an uneven and highly ridged structure with protrusions Fig. 2c^48^. SEM analysis (Fig. 2d) showed the amorphous structure of silica nanoparticles resulting from their nanoscale surface roughness^49^.

Transmission electron microscope TEM examinations Fig. 2e, f were performed to view the morphological features of the extracted SCN_NP_ fibers that were produced via the microwave radiation approach and the isolated SiO_2NP_ nanoparticles which calcined at 600 °C after treatment with HCl. The TEM images demonstrated the needle-like shape of the prepared cellulose nanocrystals, as well as the high homogeneity of their width, which ranged from 3 to 5 nm. The TEM images of the silica nanoparticles showed that the SiO_2NP_ nanoparticles were spherical, with a regular and homogeneous morphology and a diameter ranging from 6.65 to 7.68 nm; they were also somewhat agglomerated.

EDX was performed to compare the elemental composition of RH, SCN_NP_ produced by APS with microwave irradiation and SiO_2NP_, as shown in Fig. 2g, h, and i. The EDX results indicated a high proportion of silicon in the SiO_2NP_ and SCN_NP_ samples, reaching 44.34 and 19.87 wt %, respectively. In contrast, the RH fibers used to extract the SCN_NP_ contained only 5.67 wt % silicon. This highlights the effectiveness of APS in preserving the silica content during the isolation of SCN_NP_ under microwave irradiation. Furthermore, the isolated silica, primarily composed of silicon and oxygen, exhibits great purity of SiO_2NP_.

#### Bet surface area

The pore size and specific surface area were determined using the Brunauer–Emmett–Teller (BET) method. The textural and structural properties, including BET surface area (394 m^2^/g), average pore radius (7.71 nm), mean diameter (281.9 nm), and total pore volume (1.52 cc/g) demonstrate that the prepared silica possessed a high BET surface area. According to type IV isothermal adsorption–desorption curves with an H_1_-type hysteresis loop shown in Fig. 2j, the SiO_2NP_ product was primarily mesoporous^50^.

### Characterization of modified paper sheets

#### FTIR evaluation

**Fig. 3:**
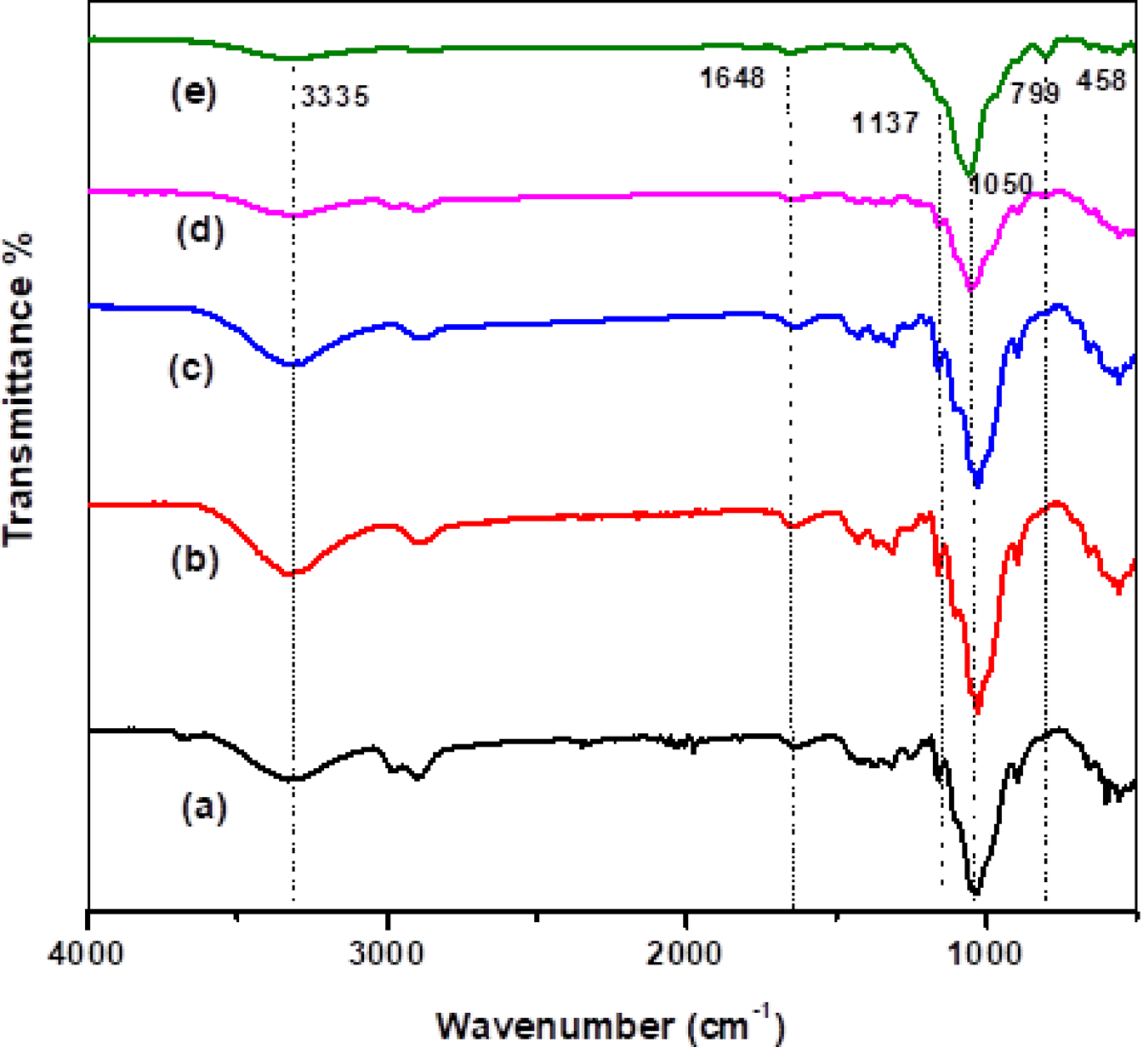
FTIR for (**a**) untreated and treated paper sheets by (**b**) CPAM, (**c**) CPAM/SCN_NP_, (**d**) CPAM/SiO_2NP_/SCN_NP_ and (**e**) CPAM/SiO_2NP_.

Paper sheets’ cellulosic fibers and the CPAM/SiO_2NP_ and/or SCN_NP_ nanocomposite were examined chemically and physically using FTIR Fig. 3. The representative absorption peaks of cellulose were visible in the unaltered paper’s FTIR spectra. A peak at 1648 cm^-1^ also indicated the presence of interstitial or adsorbed water in the cellulose structure. The C-H stretching vibration was represented by absorption in the 3000–2800 cm^-1^ range, whereas the asymmetric and symmetrical deformation of the CH_2_ and C-H groups caused absorption in the 1450–1350 cm^-1^ range. Ultimately, the fingerprint of cellulose was represented by the complex and intensive absorption in the 1300–900 cm^-1^ range, mainly related to the stretching mode of the C–O–C (1050–1060 cm^-1^) and C–O (1028 cm^-1^) bonds in the cellulose framework. The positions of these bands were influenced by intramolecular and intermolecular hydrogen bonding; hence they were strongly associated with modifications in the chemical surface groups^15^. General alterations in the absorption peaks in the corresponding FTIR spectra were noted upon incorporating the CPAM/SiO_2NP_ and/or SCN_NP_ nanocomposite into the paper sheet. The broad band at 1642 cm^-1^ for the CPAM-modified paper sheets in Fig. 3b denotes the C=O stretching vibration in the -CONH_2_ bonds. Broad band at 3335 cm^-1^ represented the -OH stretching vibration in cellulose and the -NH_2_ stretching vibration of the amide group in CPAM. Peaks at around 1158 cm^-1^ were attributed to -CO in -COOH. The characteristic band at 1137 cm^-1^ in Fig. 3c, d, and e for paper sheets treated with nanoparticles (SiO_2NP_ and/or SCN_NP_) represented the formation of Si-O-Si. When paper sheets were treated with SiO_2NP_, the peak intensities at 798 and 458 cm^-1^, which are related to the SiO_4_ tetrahedron and O-Si-O deformation, respectively, became more noticeable. The characteristic bands at 1648 and 3335 cm^-1^ represented the -OH group on the surface of SiO_2_. Furthermore, a -CH_3_ bending vibration contributed to an absorption peak at 1429 cm^-1^ on paper sheets treated with SCN_NP_. Additionally, the lower intensity of the band at 900 cm^-1^, which represents the amorphous portion of the cellulose fibers, indicates that the microwave-prepared SCN_NP_ presents a high degree of crystallinity in modified paper sheets, confirming that SiO_2NP_ and/or SCN_NP_ are successfully modified the paper sheets^51^.

#### Paper’s surface morphology

**Fig. 4:**
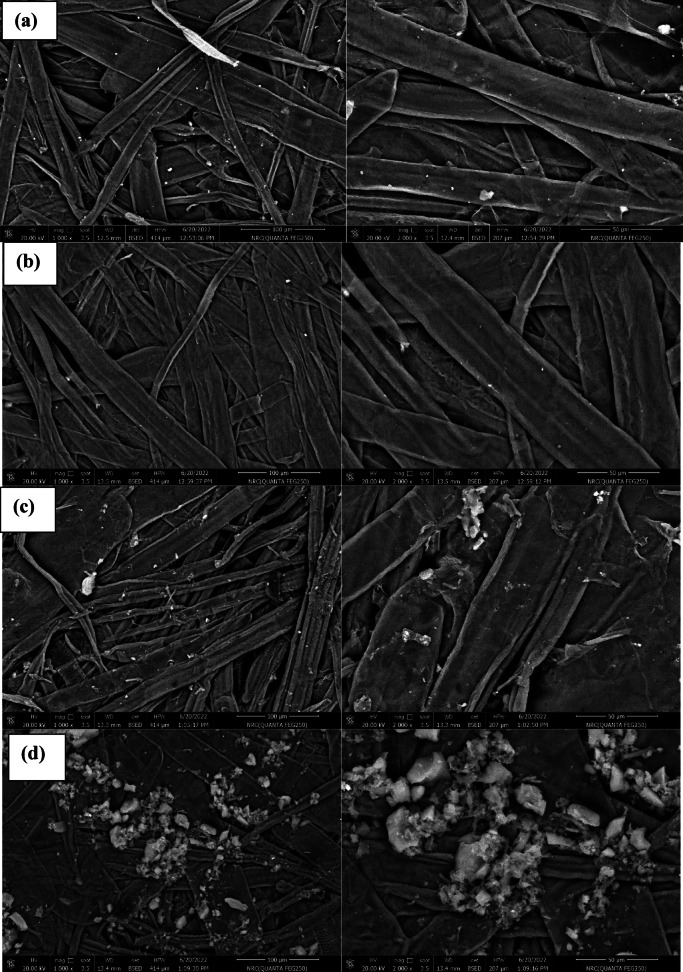

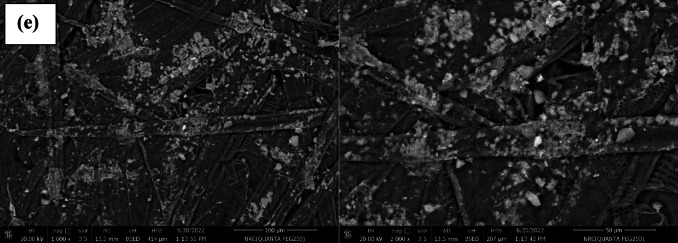
SEM for (**a**) untreated and treated paper sheets by (**b**) CPAM, (**c**) CPAM/SCN_NP_, (**d**) CPAM/SiO_2NP_/SCN_NP_ and (**e**) CPAM/SiO_2NP_.

**Fig. 5:**
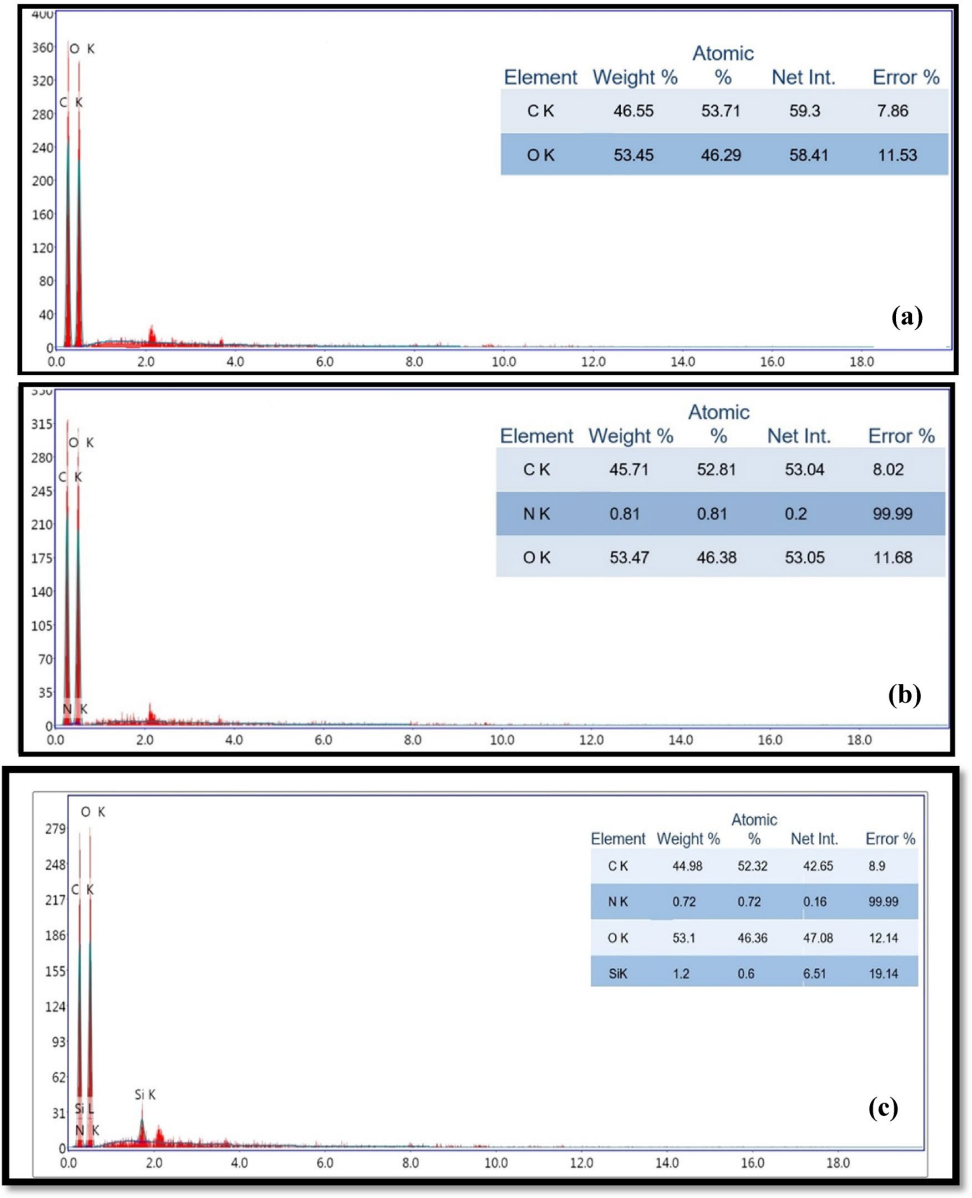

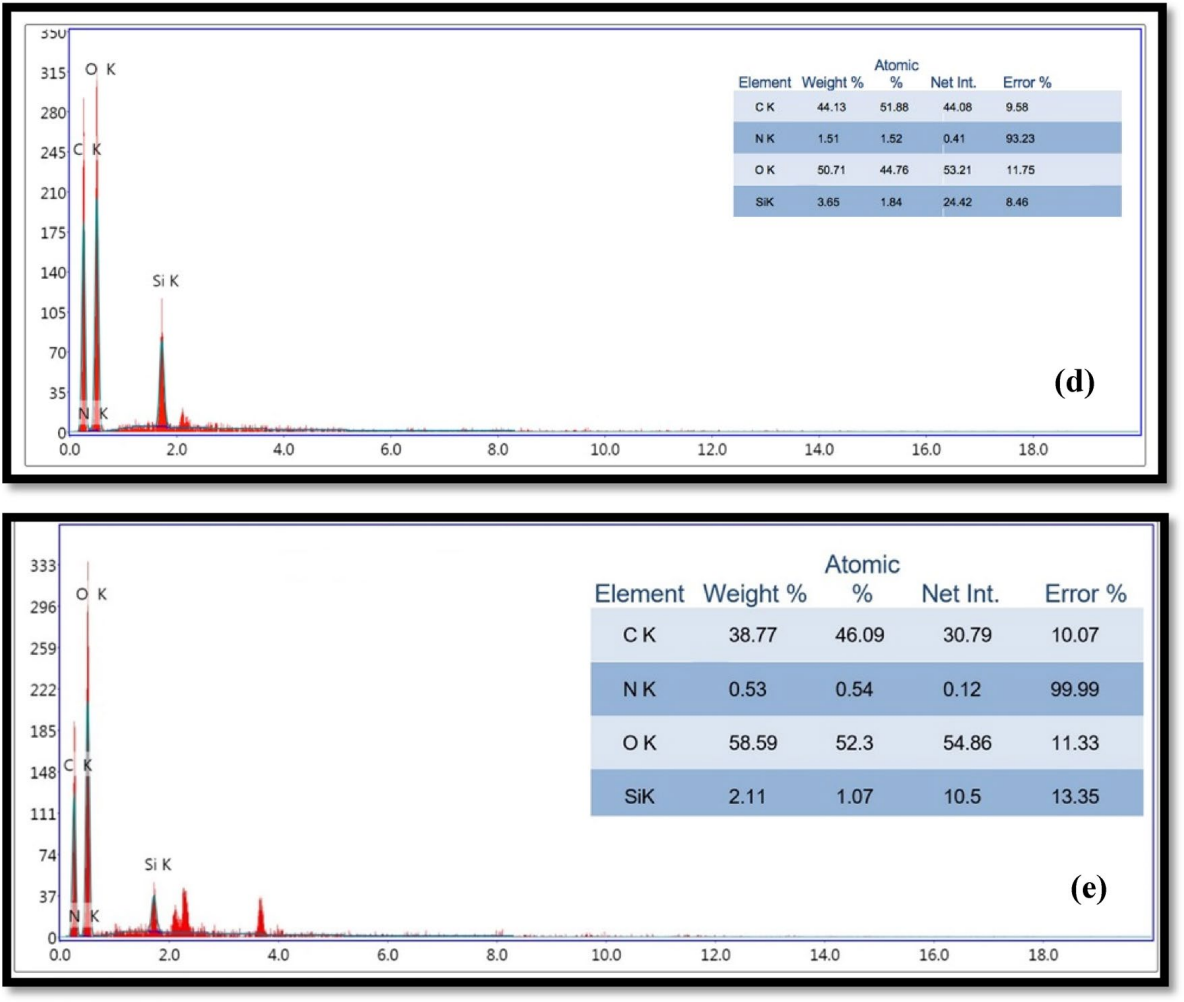
EDX for (**a**) untreated and treated paper sheets by (**b**) CPAM, (**c**) CPAM/SCN_NP_, (**d**) CPAM/ SiO_2NP_/SCN_NP_ and (**e**) CPAM/SiO_2NP_.

The surface morphology changes of both uncovered and covered paper sheets treated with various blended nanocomposites (CPAM/SiO_2NP_ and/or SCN_NP_) were illustrated in Fig. 4. The deposition of CPAM, CPAM/SCN_NP_, CPAM/SiO_2NP_/SCN_NP_, and CPAM/SiO_2NP_ significantly impacted the surface chemistry and network of cellulosic fibers. SEM analysis of the modified paper sheets revealed that CPAM formed a lamellar structure, enhancing the surface properties of the paper sheets. Additionally, the coating improved fiber dispersion within the paper matrix and the chemical compatibility of the coated nanoparticles by promoting better adhesion and bonding to the fiber surfaces. As observed, the fibers were more uniformly distributed in the coated paper sheets compared to the uncoated ones, which exhibited a random distribution. Paper sheets treated with CPAM/SiO_2NP_ appeared more homogeneous than those modified with CPAM/SiO_2NP_/SCN_NP_. The coating materials are chemically cross-linked with the cellulose fibers and were distributed both on the surface and within the fibers. Moreover, the nanoparticles tended to aggregate within the fiber-based matrices when the CPAM/SiO_2NP_/SCN_NP_ nanocomposite was used to treat the paper sheets^24^. EDAX analysis Fig. 5a–e was used to detect the elements on the modified paper surfaces. As shown in Fig. 5d, silica exhibited a prominent peak, indicating a higher proportion of silica in paper sheets covered with CPAM/SiO_2NP_/SCN_NP_.

#### Thermogravimetric analysis

**Fig. 6:**
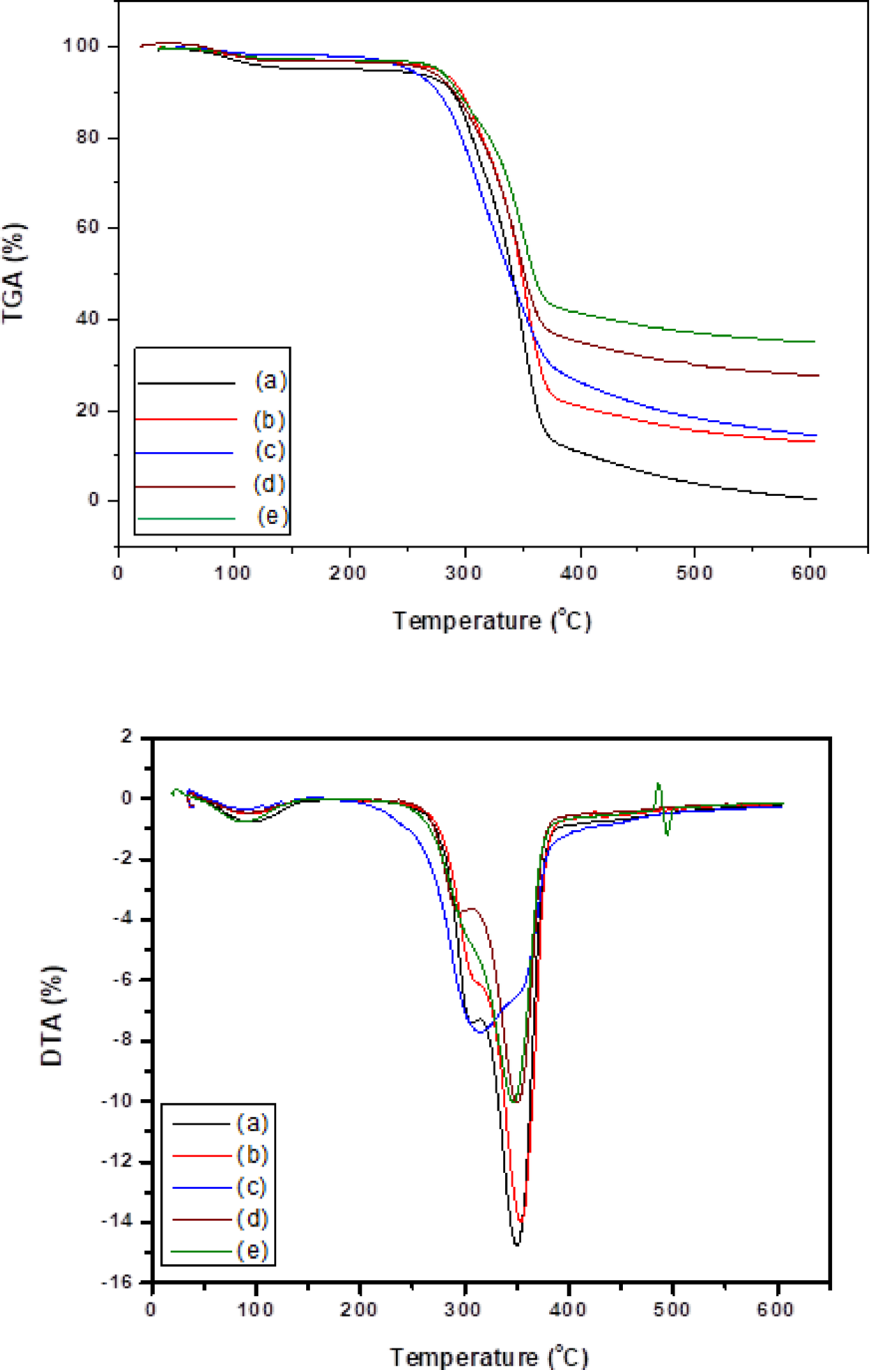
TGA and DTA for (**a**) untreated and treated paper sheets by (**b**) CPAM, (**c**) CPAM/SCN_NP_, (**d**) CPAM/ SiO_2NP_/SCN_NP_ and (**e**) CPAM/SiO_2NP_.

TGA and DTA are effective systems for examining the thermal degradation behavior of specific materials and quantifying their mass loss. Assessing how coated fillers affect a paper’s ability to maintain thermal stability under high temperatures during transfer processes and ultraviolet radiation is crucial. The decomposition curves and thermal decomposition parameters for unmodified paper sheets (S_0_) and those modified with CPAM (S_1_), CPAM/SCN_NP_ (S_2_), CPAM/SiO_2NP_/SCN_NP_ (S_6_), and CPAM/SiO_2NP_ (S_10_) mixtures, are generally displayed in Fig. 6 and Table 2. The figure clearly illustrates a three-step breakdown process, consistent with previous findings. These three phases were identified as: drying (40–150 °C), organic volatile matter elimination (215–350 °C), and carbonaceous char combustion (350–690 °C)^32^.

**Table 2:** TGA and DTA data for paper sheets treated with CPMA, CPAM/SCN_NP_, CPAM/ SiO_2NP_-SCN_NP_ and CPAM/SiO_2NP_.

Sample	Stage	TG range (°C)	DTA peak (°C)	Mass loss (%)	RC* (Wt.%)
S_0_	I	35–146	93	5.03	-
	II	254–389	350	73.72	10.67
	III	389–599	-	99.6	0.4
S_1_	I	37–142	91	4.1	-
	II	252–397	353	68.14	19.71
	III	397–602	-	87.27	12.73
S_2_	I	35–123	81	2.08	-
	II	193–405	313	60.18	24.64
	III	405–601	-	85.22	14.78
S_6_	I	37–141	87	4.02	-
	II	235–397	346	54.27	34.91
	III	397–609	-	71.66	28.34
S_10_	I	36–153	90	3.3	-
	II	235–391	350	49.86	41.49
	III	391–602	-	64.67	35.33

RC* (wt.%) is the residual char percent at temperature corresponding to constant weight loss.

According to the TG curves for our modified samples, three phases of mass loss occurred between 35 and 602 °C for the S_0_, S_1_, S_2_, S_6_, and S_10_ paper matrices. The primary decomposition stage, attributed to absorbed water evaporation, took place at (35–146, 37–142, 35–123, 37–141, and 36–153 °C), resulting in weight loss of (5.03, 4.10, 2.08, 4.02, and 3.30%), respectively. The secondary stage occurred at (254, 252, 193, 235, and 235 °C), resulting in weight loss of (73, 68.14, 60.18, 54.27, and 49.86%), respectively. The third stage, producing maximum weight loss of (99.60, 87.27, 85.22, 71.66, and 64.67%), was achieved at approximately (389, 397, 405, 397, and 391°C), reaching completion at (602, 601, 609, and 602 °C) for S_0_, S_1_, S_2_, S_6_, and S_10_, respectively.

As demonstrated in Table 2, unloaded bagasse paper (S_0_) demonstrated lower thermal stability and a reduced capability for char formation compared to the other tested paper samples. Cellulose degradation typically proceeds via a depolymerization mechanism of glycosyl units, producing high-boiling products such as levoglucosan. At higher temperatures, levoglucosan can further decompose into lighter, combustible gases. Moreover, the inclusion of metals, pigments, fillers, or additives can significantly alter the degradation pathway, decomposition products, and thermal stability^52^. Consistent with previous results, all modified samples in this study showed a greater percentage of char production and enhanced thermal stability compared to the unloaded paper sheets. The analysis further indicates a significant relationship between the degradation temperature and the incorporation of SiO_2NP_ and SCN_NP_. From Table 2, at approximately 390 °C, S_10_ showed the highest char yield production (41.49% opposed to 10.67% of control bagasse). Moreover, the residual char at 600 °C (RC_600_ °C) was 0.40% for unloaded paper S_0_, 12.73% and 14.78% for S_1_ and S_2_, respectively. While it was 28.34% and 35.33% for S_6_ and S_10_, respectively. The higher char values observed for S_6_ and S_10_ demonstrate improved thermal stability, which correlates with the presence of silica and silica-based nanocellulose nanoparticles. These improved results are attributed to the ability of the added fillers to stabilize the bagasse paper sheets structure and shift the thermal degradation process toward less volatile and non-flammable products. Furthermore, the dehydration breakdown mechanism of bagasse paper was restricted by the presence of these fillers, thus minimizing the depolymerization of cellulose to levoglucosan and promoting the formation of carbonaceous char (CO_2_, CO, H_2_O, and solid char)^53^.

The DTG curves revealed two stages of thermal degradation for both untreated paper sheets and those treated with CPAM, CPAM/SCN_NP_, CPAM/SiO_2NP_/SCN_NP_, and CPAM/SiO_2NP_. The first decomposition peaks for S_0_, S_1_, S_2_, S_6_, and S_10_, occurred at 93, 91, 81, 87, and 90 °C, respectively. The second DTG decomposition temperatures for the modified and unmodified paper sheets were 350, 353, 313, 346, and 350 °C, respectively. The findings indicated that the addition of fillers improved the thermal stability and stabilization of the bagasse structure. Based on the thermogravimetric data, the modified papers could be employed for UV protection in packaging applications and as multifunctional heat transfer printing papers for polyester sublimation printing.

### Strength properties of paper sheets coated with (CPAM/ SiO_2NP_ and/or SCN_NP_)

**Fig. 7:**
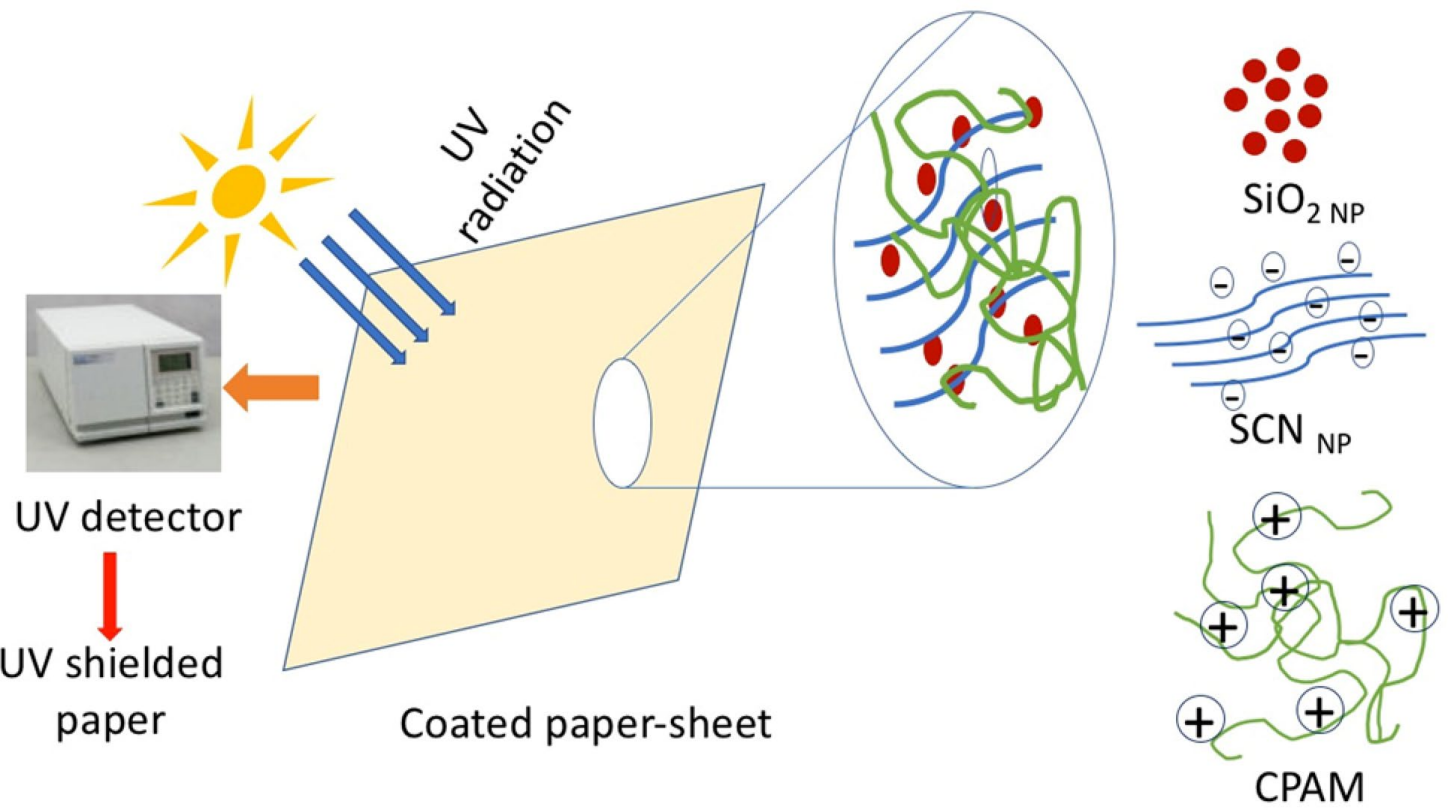
Schematic mechanism of CPAM with SCN_NP_ and SiO_2NP_.

The relationship between paper thickness and grammage is represented by the bulk density of paper sheets. Higher bulk-density papers are typically opaque, light, airy, and thick. As anticipated, the application of (CPAM/SiO_2NP_ and/or SCN_NP_) loading enhanced the bulk density of the modified paper sheets (Table 3). The attachment of (CPAM/SiO_2NP_ and/or SCN_NP_) nanocomposites favorably impacted the mechanical strength properties of the modified paper sheets, resulting in a notable increase in maximum load, tensile index, and breaking length. Measurements of paper sheets’ tensile strength provide specific information about their resistance to breaking under stress; this breaking strength is controlled by the cellulosic fibers’ strength, length, surface area, and consequently their bonding strength. Regarding the tensile index, a gauge of a paper’s intrinsic strength, it increased to 0.27 and 0.29 KN.m/g, when paper sheets modified with 0.5% SCN_NP_ mixed with 0.5% or 1% SiO_2NP_ nanocomposite, S_5_, and S_6_, respectively, were used. Furthermore, adding CPAM/SiO_2NP_ improved the observed tensile index, reaching its maximum (0.33 KN.m/g) for the modified paper sheet S_9_, which contained 1% SiO_2NP_. Table 3 clearly showed that the observed improvement percentage in the measured breaking length, approximately 35, 38, and 56% for S_5_, S_6_, and S_9_, respectively, was caused by the presence of the (CPAM/SiO_2NP_ and/or SCN_NP_) nanocomposite. According to the experimental results, paper sheets treated with SiO_2NP_/ SCN_NP_ often demonstrated better mechanical performance than untreated ones. CPAM is a positively charged polyelectrolyte with a high molecular weight (≈ 1.106). When the CPAM/SiO_2NP_ suspension was blended with nanocellulose fiber suspension, the CPAM-covered SiO_2NP_ and their assemblies were electrostatically attracted to the cellulose fibers, causing the nanocomposite to adsorb and be retained within the cellulose matrix of the paper. This explains the strong performance of modified paper sheets. The cellulose interface adsorption will prevent SiO_2NP_ assemblies from moving and help maintain the order and assembly structure of the nanoparticles^23^. Additionally, the surface of SiO_2NP_’s has numerous hydroxyl groups that can form hydrogen bonds with the hydroxyl groups in the cellulose matrix, increasing cellulosic fiber adhesion and, consequently, fiber-to-fiber bonding^54^. The schematic mechanism of CPAM with SiO_2NP_ and SCN_NP_ is represented in Fig. 7.

**Table 3:** Strength properties for paper sheets modified with (CPAM/ SiO_2NP_ and/or SCN_NP_).

Sample	Bulk Density (Kg/m^3^)		Maximum Load (N)		Tensile Stiffness (N/m)		Breaking Length (m)		Tensile Index (KN m/g)		Stiffness (N/m)		Young’s Modulus (MPa)	
	Average	STDEV	Average	STDEV	Average	STDEV	Average	STDEV	Average	STDEV	Average	STDEV	Average	STDEV
S_0_	554.92	29.28	40.64	9.13	1.03E+07	1.54E+06	2929.05	747.76	0.17	0.05	55837.32	23639.51	1781.41	815.16
S_1_	647.91	14.46	40.91	16.33	1.11E+07	1.49E+06	3520.76	1174.64	0.23	0.08	60567.87	10108.26	1891.88	337.07
S_2_	645.13	2.44	45.21	5.87	1.20E+07	9.74E+05	3775.60	422.22	0.27	0.02	74088.31	9391.70	2205.19	276.23
S_3_	685.76	1.72	51.05	9.47	1.36E+07	1.88E+06	3449.64	729.14	0.26	0.05	64311.57	9896.95	2473.52	380.65
S_4_	667.38	2.20	51.87	6.97	1.44E+07	1.61E+06	3771.00	514.98	0.26	0.04	69177.78	3598.45	2470.63	128.52
S_5_	681.66	2.79	54.51	7.35	1.47E+07	2.70E+06	3973.73	531.24	0.27	0.04	77367.18	3764.54	2865.45	139.43
S_6_	662.19	3.39	55.49	5.32	1.90E+07	1.36E+06	4071.09	405.18	0.29	0.03	92950.54	6933.46	2646.01	247.62
S_7_	657.74	3.29	52.49	7.76	1.35E+07	1.43E+06	3744.74	569.32	0.25	0.04	71903.95	7465.28	2479.45	257.42
S_8_	662.98	1.61	51.34	10.72	1.53E+07	2.53E+06	3761.72	785.03	0.26	0.05	70739.09	8685.73	2526.40	310.20
S_9_	677.33	1.11	61.58	8.38	1.56E+07	1.13E+06	4579.71	624.98	0.33	0.05	82612.49	2544.50	3059.72	94.24
S_10_	651.36	19.52	49.43	8.33	1.50E+07	2.85E+06	2937.04	536.36	0.20	0.04	65497.16	6863.48	2258.52	236.67

### Barrier properties of paper sheets coated with (CPAM/ SiO_2NP_ and/or SCN_NP_)

#### Water vapor permeability

Other measurements were performed for the modified paper’s water vapor permeability (WVP) and compared to those of the unmodified material. Coated paper sheets were examined for WVP, which was measured as the amount of water vapor that permeates a paper matrix per unit area and time under specific circumstances. As shown in Table 4, the treatment of paper sheets with SiO_2NP_ and/or SCN_NP_ induced a reduction in WVP, compared with the control sample.

**Table 4:** Water vapor permeability WVP and Oil resistance measurements for modified paper sheets.

Sample	WVP (g m^−1^ Kpa^−1^ h^−1^)		Time (s)	
	Average	STDEV(± s)	Average	STDEV(± s)
S0	1.70E-05	7.18E-06	300	2
S1	1.61E-05	9.46E-06	660	3
S2	1.92E-05	6.53E-06	900	1.5
S3	1.47E-05	5.43E-06	1200	2
S4	1.60E-05	6.98E-06	1380	1
S5	1.58E-05	7.86E-06	1770	5
S6	1.50E-05	4.33E-06	+ 1800	4
S7	1.58E-05	8.53E-06	1440	1
S8	1.51E-05	9.49E-06	+ 1800	2
S9	1.46E-05	5.89E-06	1745	2
S10	1.57E-05	7.00E-06	+ 1800	1.5

The results demonstrated that the modified paper sheets exhibited decreased hydrophilic properties, which limited the amount of water that could pass through the paper network. The blending of CPAM with SiO_2NP_ and/or SCN_NP_ likely resulted in the formation of a homogenous network on the paper sheets, which significantly reduces the voids in the paper matrix, producing a strong and rough surface and improving water vapor resistance. This reduction in porosity of the fiber matrix could explain the observed decrease in WVP and was confirmed by SEM characterization^52^. In addition to mechanical strength, low WVP is a necessary characteristic for food packaging to preserve the contents for an extended time.

#### Oil resistance

As shown in Table 4, coated paper sheets showed superior oil resistance (660–1800 + s) compared to the control paper sheets (300 s). Specifically, the S_5_ (CPAM/1%SiO_2NP_/0.5%SCN_NP_), S_6_ (CPAM/3%SiO_2NP_/0.5%SCN_NP_), S_8_ (CPAM/0.5%SiO_2NP_), and S_10_ (CPAM/3%SiO_2NP_) coated sheets demonstrated oil resistance of approximately 1800s, which meets the requirements for printing applications. The initially large pore size in the paper indicates a relative lack of pores in the paper matrix, contributing to the development of oil resistance. This compact structure can prevent oil from penetrating through capillaries; conversely, larger pore diameters facilitate oil flow through the paper matrix’s network. The presence of SiO_2NP_ in rice husk-derived silica nanoparticles and silica-based nanocellulose increases the tortuosity of the network for oil molecules, slowing their migration and penetration through the paper surface and helping fill the gaps between the cellulosic fibers. SEM reveals the aggregation of SiO_2NP_ and SCN_NP_ on the surface, further preventing oil from reaching the substrate. This strategy shows promise for printing applications requiring superior barrier features^24^.

#### UPF measurements for modified paper sheets

**Fig. 8:**
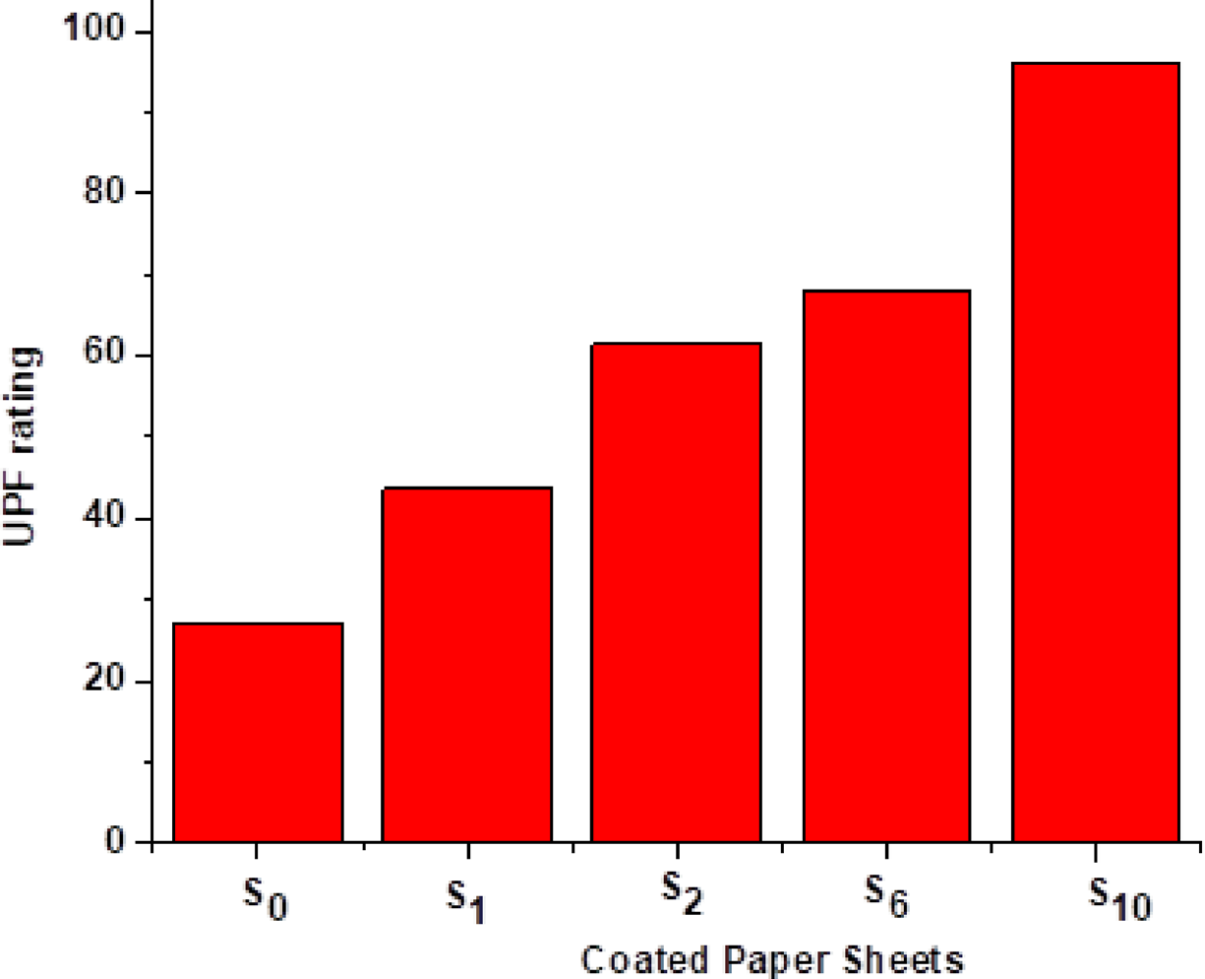
Ultraviolet protection factor (UPF) rating for modified paper sheets.

Ultraviolet Protection Factor (UPF) values are categorized as poor (below 20), acceptable (between 20 and 29), very good (between 30 and 40), and excellent (beyond 40), as seen in Table 5. The findings indicated that while modified paper sheets possess high UV protection properties due to alterations in the paper sheet matrix, untreated paper sheets exhibited low UV protection. Additionally, utilizing (CPAM/SiO_2NP_ and/or SCN_NP_) nanocomposites enhanced the UV protection of paper sheets to the maximum levels, with CPAM/SiO_2NP_ S_10_-coated paper sheets showed remarkable improvements in protection values (96.20 UPF rating and blocked 98% of UV radiation) Fig. 8. This improvement was likely caused by potential crosslinking between cellulose fibers in paper sheets and (CPAM, SCN_NP_, and SiO_2NP_) via surface hydroxyl groups, as demonstrated by previous investigations^28,55^. Both photochemical reactions and free radicals can oxidize lipids, break down proteins, destroy antioxidants, alter color and substance, and create unwanted flavorings and odors. Therefore, the group has a lone pair of electrons or π bonds that can absorb the UV wavelengths, which include the UV and visible light ranges from 200 to 800 nm. Several biopolymer-based films, such as cellulose, lignin, gelatine, and chitosan, can also absorb substantial quantities of UV radiation, depending on their structural arrangements and composition. These biopolymers frequently include functional groups like carbonyl, hydroxyl, amino, and aromatic groups that have UV screening properties. The effectiveness of a biopolymer film’s UV screening can be increased by raising the concentrations of specific functional groups in the film^56^. Exceptional UV light absorption has been recorded for various kinds of metal oxides, including ZnO, TiO_2_, and CeO_2_. Incorporating TiO_2_ NPs into CMC/guanidinylated chitosan films, for example, has been shown to block 98% of UV light^57,58^. The treated samples exhibited lower transmittance % for both UVA and UVB radiation when compared to the blank sample, as shown in Table 6. The paper sheet treated with CPAM/SiO_2NP_ (S_10_) exhibited the lowest transmittance rate 3.4 and 0.8 for UVA and UVB radiation, respectively compared to S_0_ sample. Xia, Zhou, et al. (2021) demonstrated an improvement in UV shielding characteristics for a cellulose matrix made from wasted corrugated boxes. The results revealed that the cellulose-based sheets produced by water and ethanol treatment recorded transmittance percentages of 34.68 and 33.42 for UVA and 13.9 and 14.14 for UVB, respectively, compared to the blank of 86.08 and 79.77^59^. Ahmed, Adak, et al. (2019) fabricated lyocell-based nanocellulose NC/graphene oxide GO nanocomposite film sheets. The quantified UVA and UVB transmittance % for NC/GO film were 76.75% and 63.66%, respectively, indicating that, it has a low UV protection ability in both UVA and UVB radiation zones^60^. The carboxyl, hydroxyl, and carbonyl functional groups in SCN_NP_ and SiO_2NP_ nanoparticles are essential to their UV-shielding capabilities and are known to enhance their efficacy^61,62^. Generally, CPAM/SiO_2NP_-coated paper sheets exhibited higher UV resistance than other samples. Figure 7 displays a graphic representation of a paper sheet coated with a CPAM/SiO_2NP_/SCN_NP_ nanocomposite for UV protection. In our study, the functionalized CPAM/SiO_2NP_ and/or SCN_NP_ nanocomposite was applied not only as a transfer paper used in textile printing applications but also as a multifunctional coated paper sheet for UV protection in the packaging fields.

**Table 5:** Classifications and grades of UPF.

UPF rating	Protection category	% UV radiation blocked
> 15	Poor	–
15–24	Good	93.3–95.9
25–39	Very good	96.0–97.4
40–50+	Excellent	97.5–98+

**Table 6:** UVA and UVB transmittance value of paper sheets.

Sample	UVA (280–320 nm)	UVB (320–400 nm)
S_0_	10.3	2.8
S_1_	7.1	1.7
S_2_	5.3	1.2
S_6_	4.4	1.2
S_10_	3.4	0.8

### Color properties of printed paper

Since cellulose paper has no affinity for dispersed dyes, it is the primary choice as a carrier for these dyes in sublimation transfer printing. To avoid a mottled effect caused by varying thermal insulation of the paper when in contact with the heating element and non-homogeneous paste adsorbents, a high-quality with a uniform-structure is a basic requirement for an accurate and high-design transfer from the applied printed paper. This paper must be capable of releasing non-absorbed dye and also be strong enough to withstand the action of heat and pressure^63,64^.

The percentage of paste uptake on different treated sheets is shown in Table 7. As the concentration of silica nanoparticles in the treatment process increases, the dye uptake percentage decreases. The presence of SiO_2NP_ helps fill the gaps in paper structure. This allows the water content of the printing paste penetrates the inner layer, keeping dye paste adhered to the surface. Furthermore, the SiO_2NP_ imparts the required level of hydrophobicity to control dye paste adsorption and avoid a greater tendency to absorb water, resulting in poor printing properties. Table 7 also showed the color properties of printed paper sheets using disperse dye paste, indicating that all the treated paper samples had improved or comparable color properties to that of commercial transfer paper (lower L value indicates darker color shade)^32,34,65^.

**Table 7:** The Past uptake percentage, and CIELAB color parameters of printed modified paper sheets using silk screen technique, Papers λ_max_ = 530 nm.

Sample	% of past uptake	L*	a*	b*
Commercial transfer paper	4.05	39.91	37.32	5.43
S_0_	4.87	38.31	36.72	7.69
S_1_	3.01	35.79	32.81	9.01
S_2_	3.56	38.54	36.43	7.14
S_3_	3.76	36.50	33.20	8.07
S_4_	4.45	37.06	34.49	8.31
S_5_	2.67	38.10	36.33	7.87
S_6_	3.16	35.74	33.85	9.41
S_7_	4.26	37.39	35.08	7.98
S_8_	2.12	37.11	34.87	7.48
S_9_	2.34	38.25	37.02	7.53
S_10_	2.23	36.51	33.67	8.90

**Fig. 9:**
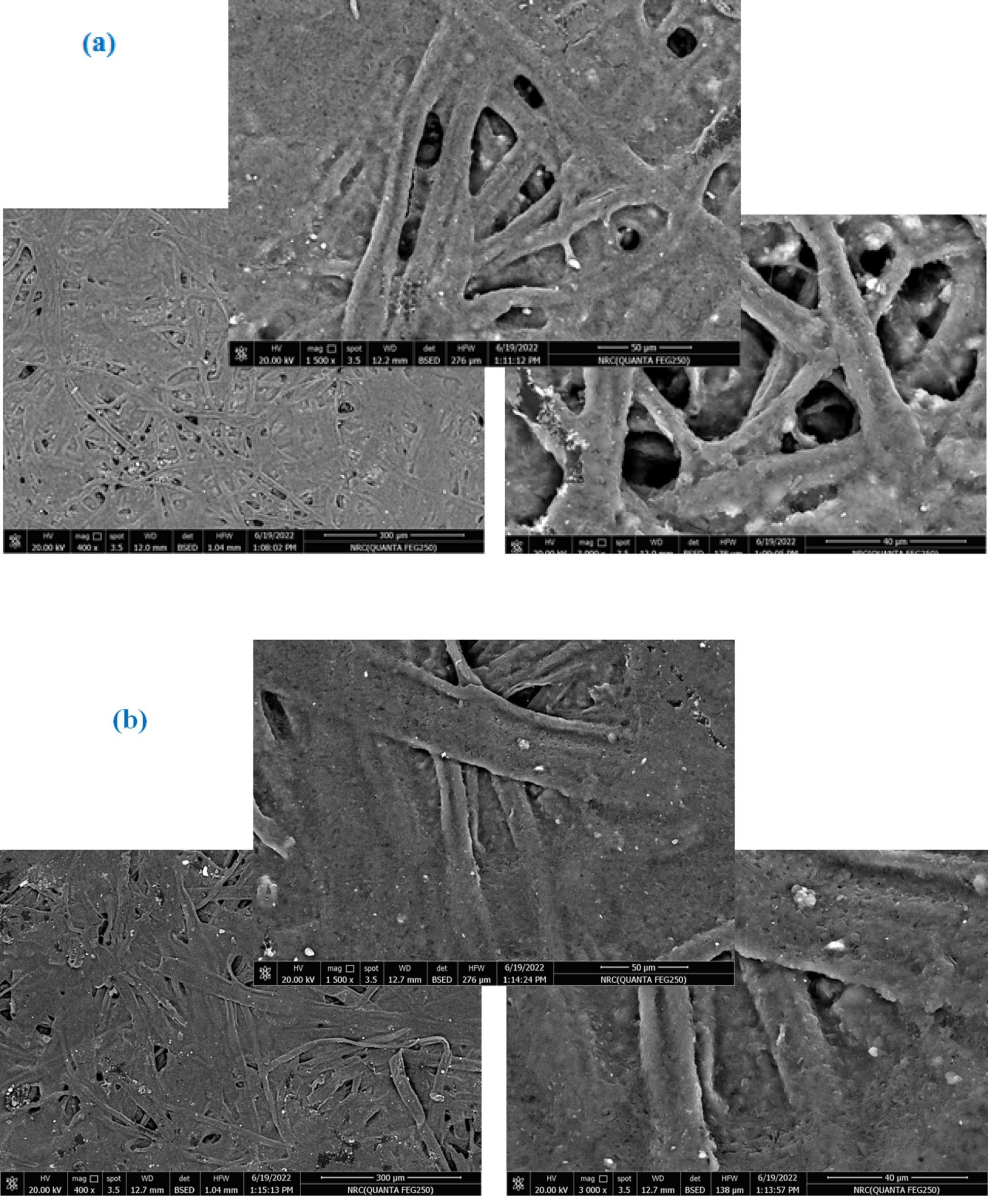

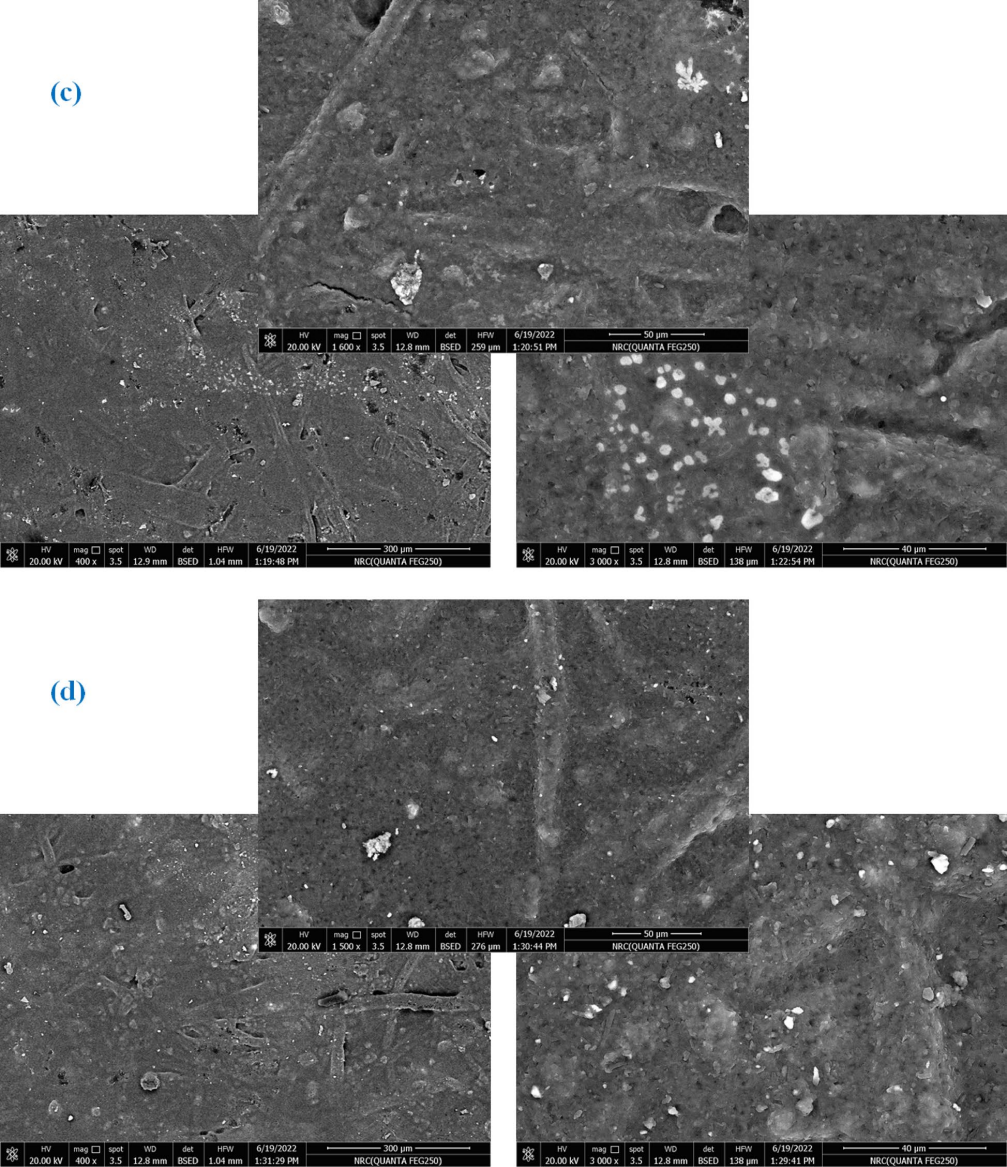
SEM images of printed commercial transfer paper (**a**), printed untreated paper (S_0_) (**b**), printed paper treated with CPAM/0.5% SiO_2NP_ (S_8_) (**c**), and printed paper treated with CPAM/3% SiO_2NP_ (S_10_) (**d**).

Furthermore, the microscopic surface profile, as revealed by SEM images, showed that the printed paper treated with CPAM/0.5% SiO_2NP_ (S_8_) and CPAM/3% SiO_2NP_ (S_10_) displayed a more homogeneous and uniform surface, featuring fewer gaps and surface imperfections when compared to commercial or untreated paper sheets. Additionally, the upper layer containing dye paste appeared smoother, enhancing the prints’ consistency and clarity, as illustrated in Fig. 9^63^. Consequently, S_8_ and S_10_ paper sheets are expected to exhibit improved color transfer production when utilized as transfer paper for sublimation polyester printing, as demonstrated in the next section.

### Color depth and fastness properties of polyester fabric printed by treated paper via sublimation transfer technique

**Fig. 10:**
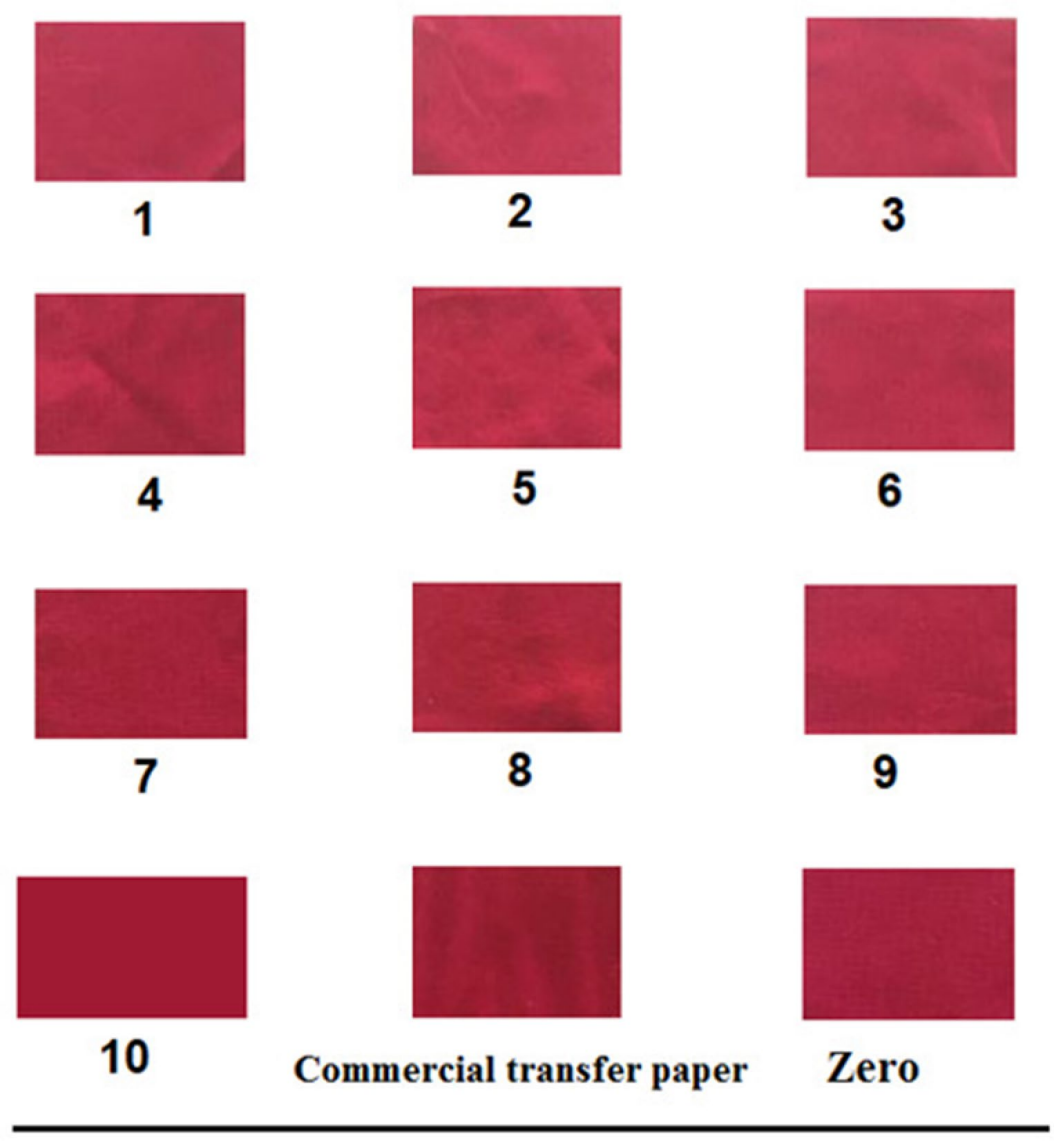
Photographic image for printed polyester fabrics by treated paper via heat transfer technique for (S_1_), (S_2_), (S_3_), (S_4_), (S_5_), (S_6_), (S_7_), (S_8_), (S_9_), (S_10_), commercial and (S_0_) untreated paper, respectively.

The color depth represents the actual dye content on the printed fabric, i.e. K/S. Table 8 shows that the K/S and CIE lab color parameters for polyester fabrics printed using the sublimation heat transfer technique with both former-treated and commercial paper sheets. The results indicate that the type of treatment, as well as the time and temperature of the transfer process, are the key factors in controlling the color depth and the quality of dye preserved by the fabrics. Increasing the transfer temperature and/or time, resulted in more dye transfer, consequently increasing the color strength (K/S value) of the printed fabrics due to the increased vapor pressure of the applied dye. Furthermore, to improve the dye affinity for the fiber and prevent dye retention on the paper surface^32,66^, the affinity of the dye vapor for the paper filter, coating, treatment, and printing paste thickener should be minimized. Consequently, the best color yield improvements were obtained at 210 °C for 60 s, which were established as the optimum conditions, as shown in Table 8 and Fig. 10.

**Table 8:** The K/S and CIE lab color parameters of heat transfer printed polyester fabrics using modified screen-printed paper sheets at different transfer conditions, λ_max_ = 525 nm.

Temperature (°C)	170		190		210				
Time (sec.)	30	60	30	60	30	60			
Sample						K/S	b*	a*	L*
Commercial transfer paper	0.40	4.29	3.26	7.11	6.09	18.66	38.37	56.02	14.20
S_0_	0.59	2.59	1.59	5.71	3.62	17.39	40.08	59.11	7.17
S_1_	0.49	3.96	2.94	11.04	5.05	19.35	38.06	58.17	10.99
S_2_	0.42	2.73	1.72	8.41	4.10	18.30	38.29	57.85	10.58
S_3_	0.48	3.61	2.59	8.65	5.94	19.86	36.78	56.44	13.13
S_4_	0.49	3.16	2.15	9.24	4.92	19.89	38.03	58.31	11.75
S_5_	0.38	2.73	1.72	9.47	3.59	19.12	38.97	59.11	10.05
S_6_	0.46	4.17	3.95	7.71	7.91	19.13	38.48	58.18	11.15
S_7_	0.77	3.00	2.00	10.78	5.60	19.66	37.45	57.42	12.42
S_8_	0.64	3.52	2.51	9.58	5.48	20.54	36.78	56.67	13.29
S_9_	0.44	4.62	2.60	6.93	4.23	19.29	38.46	58.38	10.37
S_10_	0.67	4.29	1.54	8.50	4.41	20.47	36.69	58.42	12.36

The effect of paper treatment on the ease of dye release and color reproduction during polyester transfer printing was investigated. It was noticed that all treated paper samples exhibited improved dye release ability with sheets treated with S_8_ (CPAM/0.5% SiO_2NP_) and S_10_ (CPAM/3%SiO_2NP_). This could be attributed to the silica nanoparticles’ paper treatment, which facilitates the release of the highest percentages of dye from their surface. As mentioned previously, SiO_2NP_ nanoparticles fill the gaps in the fiber matrix, reducing the diffusion of the printing paste to inner layer and localizing dye particles on the paper surface. This localization helps achieve the best possible color density on the prints, reducing dye waste and increasing paper efficacy. This observation also aligns with the reported results of paper dye uptake^67^. Regarding the fastness properties illustrated in Table 9, all printed fabrics exhibited excellent fastness.

**Table 9:** Fastness Properties of transfer printed polyester fabrics using modified screen-printed paper sheets at 210 °C and 60 s.

Samples	Light	Washing fastness			Perspiration					
					Acidic			Alkaline		
		St		Alt	St		Alt	St		Alt
		Cotton	Wool		Cotton	Wool		Cotton	Wool	
Commercial transfer paper	6	5	5	5	4-5	4-5	4-5	4-5	4-5	4-5
S0	6	5	5	5	4-5	4-5	4-5	4-5	4-5	4-5
S8	6	5	5	5	4-5	4-5	4-5	4-5	4-5	4-5
S10	6	5	5	5	4-5	4-5	4-5	4-5	4-5	4-5

St. — Staining; Alt. — Alteration.

**Fig. 11:**
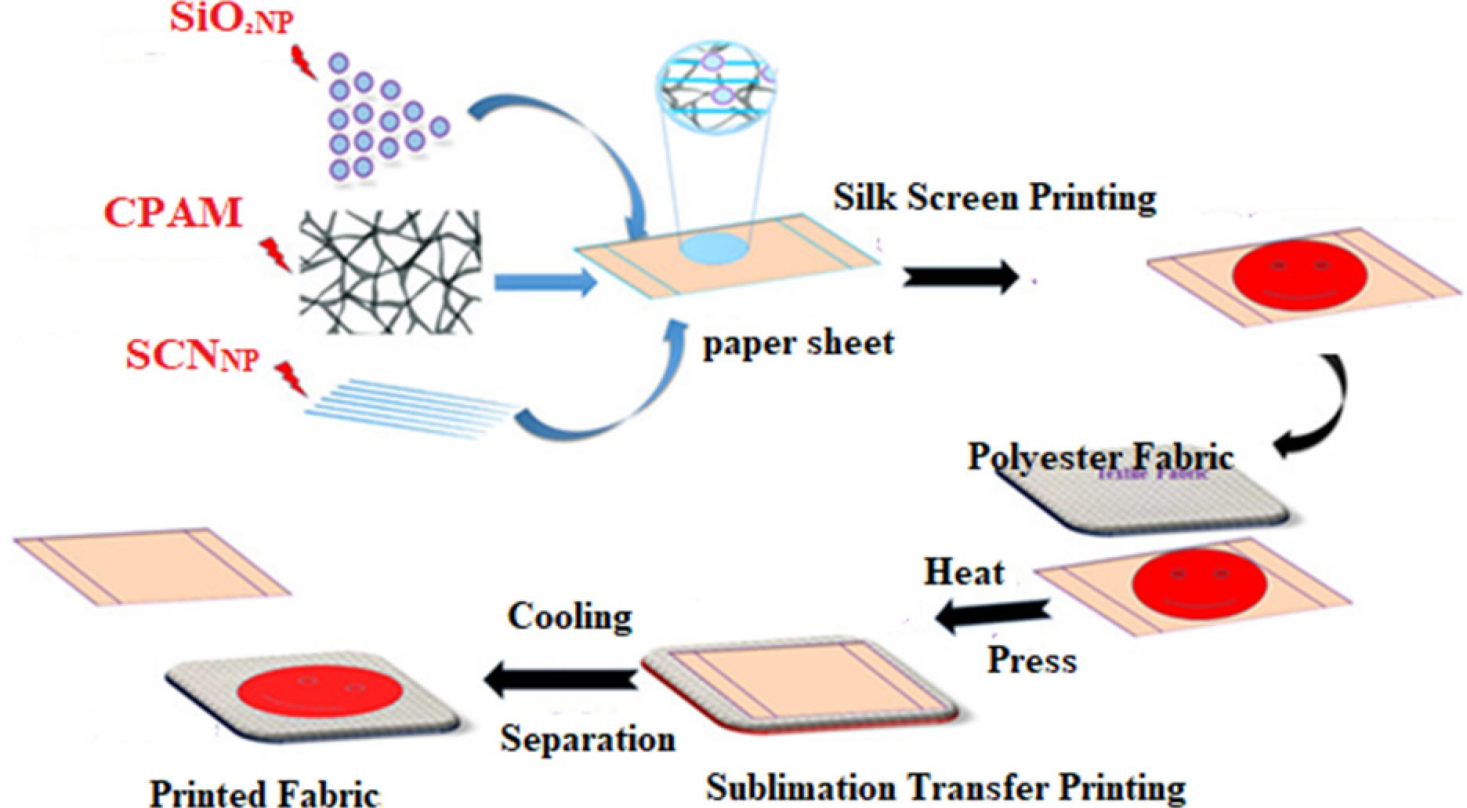
Graphical representation for heat transfer printing paper.

Additional constructive outcomes have been obtained from the reutilization of modified printed paper (under optimal conditions) for an additional printing run to evaluate the efficiency (durability) of the treated paper sheets for multiple transfer processes, aiming to reduce costs and preserve the environment. Table 10 displays the color strength and color parameters of polyester fabric printed by heat transfer technique using treated paper sheets printed with disperse dye, previously used in the heat transfer printing process at 210 °C and 60 s, for a second printing run under the same conditions. In this second transfer run, all the treated paper sheets demonstrated comparable color depth. The paper samples S_8_ (CPAM/0.5% SCN_NP_) and S_10_ (CPAM/3% SiO_2NP_) demonstrated the highest color depth transfer to polyester fabrics. This could be attributed to the ability of these treatments to improve the properties of the paper sheets by forming extra hydrogen bonds between the fibers, filing the gaps, which further act as a binder in the paper structure. This provides protection for the paper fibers from deterioration or destruction under severe transfer conditions^68^. Figure 11 presents a graphic representation of the heat transfer printing technique involving multifunctional, scalable, coated paper sheets (CPAM/SiO_2NP_ and/or SCN_NP_).

**Table 10:** The K/S and CIE lab color parameters of transfer printed polyester fabrics using modified screen-printed paper sheets at 2nd run under 210 °C, 60 s, λ_max_ = 525 nm.

Sample	K/S	L*	a*	b*
Commercial transfer paper	16.17	38.08	57.20	10.22
S_0_	16.26	39.80	57.41	6.80
S_1_	17.12	36.39	55.16	12.12
S_2_	17.22	38.18	55.90	9.31
S_3_	18.75	36.80	55.58	12.02
S_4_	18.79	36.57	55.21	12.15
S_5_	17.65	38.46	57.14	9.18
S_6_	19.98	37.23	56.58	11.24
S_7_	18.82	37.23	56.38	11.33
S_8_	19.21	35.94	54.10	11.49
S_9_	16.52	38.62	56.21	8.82
S_10_	19.43	36.84	57.62	11.33

## Conclusions

Silica-based cellulose nanocrystals (SCN_NP_) with silica content can be obtained from rice agro-waste through an economical and environmentally friendly one-step process. This method avoids conventional extraction procedures by utilizing ammonium persulfate under microwave radiation. Microwave irradiation significantly reduces the time and energy required for SCN_NP_ extraction from several hours to just a few minutes. The physicochemical and morphological characteristics of SCN_NP_ and silica nanoparticles isolated from rice husks were evaluated. A corresponding peak for the carboxyl group appeared at 1724 cm^-1^ for SCN_NP_, which can be interpreted as the -C=O valence vibration of -COOH groups. The oxidation of C_6_ primary hydroxyl groups by APS facilitates the synthesis of carboxyl groups through the interaction of cellulose fibers with free radicals created during the chemical treatment under microwave irradiation. Distinctive vibrational peaks for Si-O-Si also emerged at wavenumbers approximately 797 cm^-1^ and 464 cm^-1^. The siloxane group stretching vibrations in the produced silica nanoparticles (SiO_2NP_) were responsible for the peaks at 1096, 967, 784, and 464 cm^-1^. A peak corresponding to SiO_2_ appeared at a Bragg 2θ angle of 44.48° for RH, SCN_NP_ and SiO_2NP_. The CPAM/SiO_2NP_ and/or SCN_NP_ nanocomposites substantially functionalized paper sheets produced via bagasse waste treatment. Structural and morphological analysis of coated papers demonstrated that the SiO_2NP_/SCN_NP_ nanocomposite was distributed uniformly over the paper’s cellulose matrix. Our findings further showed that the final mechanical and barrier characteristics of the paper sheets functionalized with the SiO_2NP_ and/or SCN_NP_ nanocomposite were highly valued. Whereas, adding CPAM/SiO_2NP_ improved the observed tensile index, reaching its maximum (0.33 KN m/g) for the modified paper sheet S_9_, which contained 1% SiO_2NP_. The S_5_ (CPAM/1%SiO_2NP_/0.5%SCN_NP_), S_6_ (CPAM/3%SiO_2NP_/0.5%SCN_NP_), S_8_ (CPAM/0.5%SiO_2NP_), and S_10_ (CPAM/3%SiO_2NP_) coated sheets demonstrated oil resistance of approximately 1800s, which satisfies the requirements for printing applications. Modified paper sheets with (CPAM/SiO_2NP_ and/or SCN_NP_) nanoparticles have a multifunctional role in UV protection for packaging and as transfer paper for the polyester printing process. Coated paper containing SiO_2NP_ consumed less dye paste and produced higher color-density prints. Moreover, the (CPAM/SiO_2NP_) composite-coated papers S_8_ and S_10_ exhibited better printability, showing good stability in the second printing run.

## Data Availability

This published article contains all the data obtained or examined during this investigation.
